# *Ginkgo biloba* Extract for Intermittent Claudication in Patients with Peripheral Arterial Disease: A Systematic Review and Meta-Analysis of Randomized Controlled Trials

**DOI:** 10.3390/jcm15218606

**Published:** 2026-11-04

**Authors:** Haengjin Ohe, Jinhee Hwang, Suk Ran Kim, Seonwoo Kim, Sun Cheol Park

**Affiliations:** 1 Division of Vascular and Transplant Surgery, Department of Surgery, Seoul St. Mary’s Hospital, The Catholic University of Korea, Seoul 06591, Republic of Korea; omarch@daum.net; 2 Academic Research Service Department, LSK Global Pharma Services Co., Ltd., Seoul 04535, Republic of Korea; hwang.jinhee@lskglobal.com (J.H.); kim.seonwoo@lskglobal.com (S.K.); 3 Clinical Strategy Department, LSK Global Pharma Services Co., Ltd., Seoul 04535, Republic of Korea; kim.sukran@lskglobal.com

**Keywords:** peripheral artery disease, intermittent claudication, *Ginkgo biloba* extract, absolute claudication distance, systematic review, meta-analysis

## Abstract

**Background/Objectives**: Intermittent claudication (IC) secondary to peripheral artery disease (PAD) causes exertional leg pain and discomfort, which limits functional walking capacity. The clinical efficacy of *Ginkgo biloba* extract (GBE) as a therapeutic agent for IC is debatable. This systematic review and meta-analysis evaluated the efficacy and safety of GBE compared with a placebo in patients with IC due to PAD. **Methods**: Databases including PubMed, Embase, the Cochrane Library, and CINAHL were searched until 24 April 2026. Eligible studies included randomized, double-blind, controlled trials (RCTs) comparing placebo. Primary outcome was absolute claudication distance (ACD) at the end of the study. Secondary outcomes were initial claudication distance (ICD), ankle-brachial index (ABI), ankle pressure, and adverse events. Walking outcomes across various treadmill protocols were standardized to estimate caloric expenditure (kcal). A random-effects model was used to pool the effect estimates across trials. Risk of bias was assessed using the Cochrane risk-of-bias tool (RoB 2). **Results**: A total of 14 RCTs comprising 893 participants represent the total number randomized were included. GBE was associated with a statistically significant but modest improvement in ACD compared with placebo (mean difference [MD], 3.50 kcal; 95% CI, 0.02 to 6.98; *p* = 0.049). This benefit was significant in trials with a 24-week duration (MD, 4.67 kcal; 95% CI, 2.26 to 7.08; *p* < 0.001) but not in 12–16 weeks trials (MD, 1.35 kcal; 95% CI, −2.39 to 5.09; *p* = 0.48). No significant changes were observed for ICD or resting ABI/ankle pressure. GBE was well tolerated, with an overall adverse event incidence comparable to placebo. **Conclusions**: In patients with IC due to PAD, GBE therapy improves absolute walking performance, with a significant effect observed in trials evaluating 24 weeks of treatment. Given its favorable safety and tolerability profile, GBE can represent a clinically relevant and well-tolerated therapeutic option to support functional walking capacity, particularly for patients unable to tolerate or use conventional pharmacological agents.

## 1. Introduction

Peripheral artery disease (PAD) is primarily characterized by atherosclerotic obstruction of the lower extremity arteries. It affected an estimated 113.7 million individuals worldwide in 2021, and is a growing global health burden associated with substantial morbidity and functional impairment [1,2]. Intermittent claudication (IC) is a classic clinical manifestation in symptomatic patients with PAD, which is characterized by discomfort, pain, or fatigue in the muscles of the lower extremities and is reproducible upon exertion and relieved by rest. This symptomatic profile often restricts mobility leading to pronounced functional disability and impaired health-related quality of life [3,4,5].

Supervised exercise therapy (SET) has been established as the primary conservative therapeutic approach [3,5]. Despite strong evidence of its efficacy in improving walking performance and quality of life, the real-world implementation of SET is often hindered by significant barriers including limited patient accessibility, insufficient long-term adherence, and a requirement for substantial logistical support [6,7]. Furthermore, a considerable subset of patients with IC fail to achieve meaningful functional improvement with exercise therapy, necessitating alternative therapies, particularly pharmacotherapy, to enhance walking performance and alleviate ischemic symptoms [8].

For the pharmacological management of IC, pentoxifylline, cilostazol, and other agents such as prostaglandin analogues and serotonin 5-HT2A receptor antagonists are used in clinical practice. Among these agents, cilostazol, a phosphodiesterase III inhibitor with vasodilatory and antiplatelet properties, is the most extensively evaluated evidence-based pharmacological agent for IC treatment [9]. However, its clinical utility is substantially limited by safety concerns such as contraindications in patients with heart failure and adverse events such as headache, diarrhea, and palpitations that frequently lead to treatment discontinuation [10]. Consistent with this, a real-world retrospective study reported that approximately 20% of patients failed to adhere to cilostazol therapy in the first 3 months of treatment suggesting that tolerability challenges observed in clinical practice may be more pronounced than that reported in controlled trials [11]. Consequently, a meaningful clinical gap persists emphasizing the need for well-tolerated and efficacious alternative pharmacotherapies for long-term use. 

*Ginkgo biloba* extract (GBE), derived from the leaves of the *Ginkgo biloba* tree and used in herbal medicines for centuries, has been investigated since the 1980s as a pharmacological option for the treatment of IC, owing to its proposed mechanisms on peripheral blood flow enhancement and antioxidant activity [12]. However, its clinical efficacy remains controversial with inconsistent results across randomized placebo-controlled trials and meta-analyses. The most comprehensive evidence on the use of GBE, to date, is the 2013 Cochrane review of 14 trials with 739 participants. The authors of that study found that GBE increased absolute claudication distance (ACD) by a weighted mean of 64.5 m compared with placebo but a clinically meaningful benefit could not be confirmed largely due to small sample sizes and methodological heterogeneity [13].

Despite more randomized controlled trials being conducted, more than a decade has passed without an updated synthesis. Therefore, an updated systematic review and meta-analysis incorporating more recent trials is warranted to clarify the efficacy and safety of GBE in patients with IC due to PAD. This systematic review and meta-analysis aimed to evaluate the effects of GBE compared with a placebo on walking performance, hemodynamic status, and safety in patients with IC due to PAD.

## 2. Materials and Methods

This systematic review and meta-analysis were conducted in accordance with the Preferred Reporting Items for Systematic Reviews and Meta-Analyses (PRISMA) 2020 statement [14]. The review protocol was registered in the International Prospective Register of Systematic Reviews (PROSPERO; CRD420261424296). This review evaluated the efficacy and safety of oral GBE compared with a placebo in adults with IC due to PAD.

### 2.1. Outcomes

The primary outcome was assessment of ACD at the end of the study period. ACD was defined as the maximum walking capacity during a walking distance test until leg pain or claudication symptoms prevented further walking. Secondary outcomes included initial claudication distance (ICD), ankle-brachial index (ABI), ankle pressure, and adverse events. ICD was defined as the walking distance or time until the initial onset of claudication symptoms during the walking distance test. ACD and ICD were measured at 12–16 and 24 weeks. ABI and ankle pressure were evaluated as measures of lower extremity arterial perfusion. Adverse events included adverse reactions, side effects, and clinical or biological tolerability findings reported in individual trials. 

### 2.2. Data Collection Methods

A systematic literature search was conducted in PubMed, EMBASE, the Cochrane Library, and CINAHL on 24 April 2026. The review followed PRISMA guidelines (Supplementary Tables S1 and S2). Controlled-terms and free-text terms related to IC, PAD, peripheral arterial occlusive disease, and *Ginkgo biloba* were combined with terms related to randomized controlled trials. Language restrictions were not imposed. Detailed search strategies for each database are provided in Supplementary Table S3. The reference lists of included studies were manually searched to identify additional eligible studies. Both published and unpublished randomized trials were considered to minimize the risk of publication bias. To identify unpublished studies, manufacturers of *Ginkgo biloba* products were contacted for information on published and unpublished trials. Previous systematic reviews particularly the Cochrane review of *Ginkgo biloba* for intermittent claudication were also used to identify relevant reports and unpublished or internal trial data when applicable [13]. 

### 2.3. Eligibility Criteria

Studies were eligible if they met the following criteria: (a) Randomized controlled trials (RCTs) on GBE versus placebo in adults with IC (Fontaine stage II or Rutherford categories 1–3). (b) Adult patients aged ≥18 years (c) GBE including EGb 761, irrespective of dosage, and (d) at least one of the two predefined walking distance outcomes: ACD or ICD.

We excluded studies that included patients with advanced PAD including those with Fontaine stage III or IV disease, rest pain, ulceration, or gangrene. Patients with cardiopulmonary, orthopedic, or other conditions that could independently limit their walking capacity were also excluded. Studies combining exercise were eligible if the effect of *Ginkgo biloba* could be evaluated independently of the exercise intervention. 

### 2.4. Study Selection

**Figure 1:**
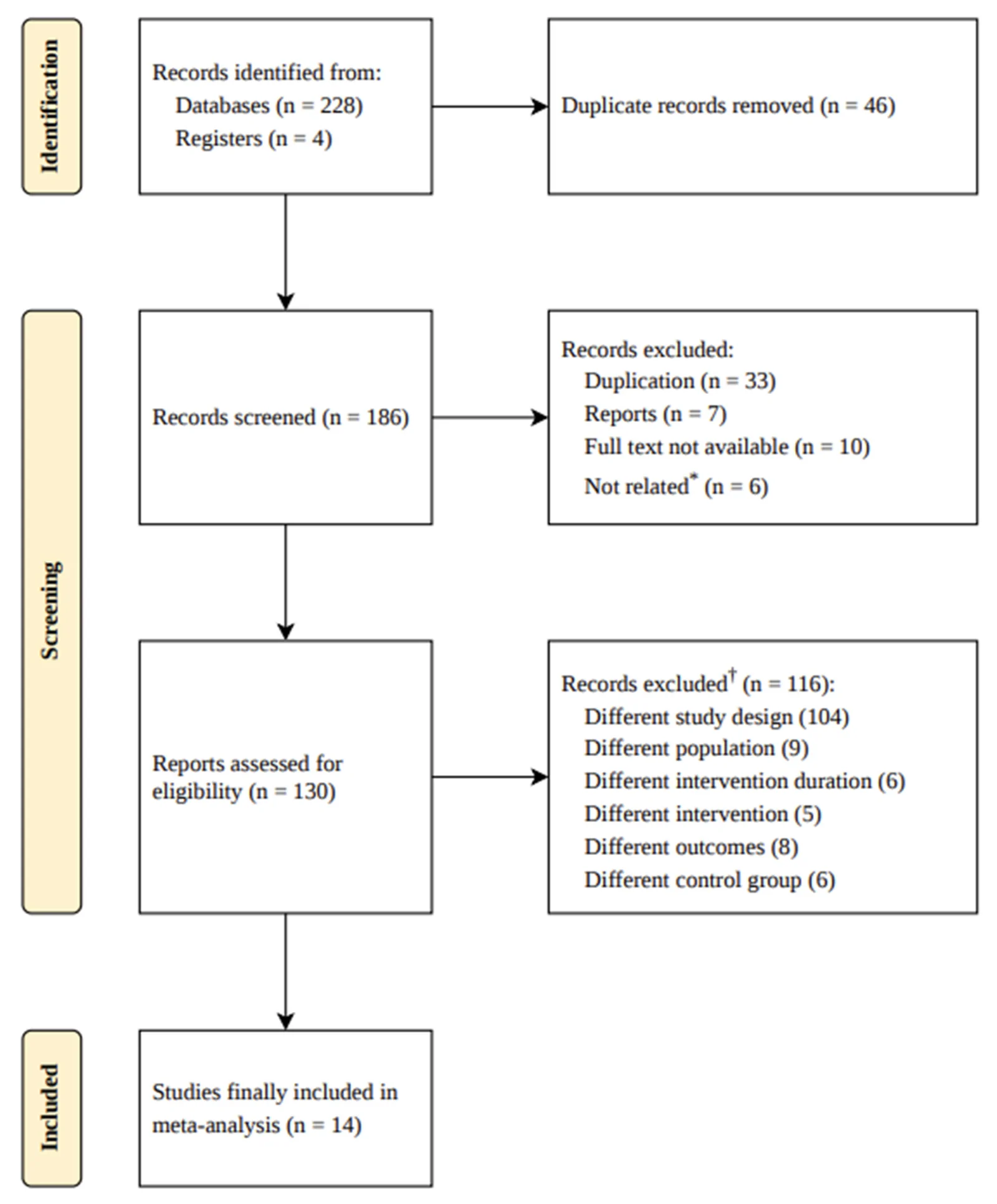
PRISMA diagram. * Studies that did not meet all predefined PICO criteria. ^†^ Some reports met more than one exclusion criterion. Reasons are therefore not mutually exclusive, and their sum (*n* = 138) exceeds the number of excluded reports (*n* = 116).

All retrieved references were imported to EndNote 2025 for reference management and initial duplicate removal. After duplicate removal, titles and abstracts identified through the systematic search were independently screened by two reviewers. Full-text reports were retrieved for studies that met or potentially met the eligibility criteria and were independently assessed by the same reviewers. Published articles, abstracts, and available unpublished or internal reports were considered during the eligibility assessment. Disagreements were resolved by discussion and consensus. Reasons for exclusion were documented during the full-text assessment. The study identification and selection processes are presented in a PRISMA flow diagram (Figure 1).

### 2.5. Data Extraction and Quality Assessment

Data was independently extracted by two reviewers using a standardized data extraction form. The extracted information included the publication year, study design, country, sample size, participant age and sex, intervention and comparator, daily GBE dose, treatment duration, and outcomes of interest. For trials previously included in the Cochrane review for which the original report was unavailable or numerical walking distance data could not be retrieved, the standardized caloric expenditure data reported in the Cochrane review including group-specific sample sizes, means, and standard deviations, were used [13]. In a recent randomized trial [15], available individual participant data were used to derive walking capacity-related outcomes. 

Risk of bias was assessed using the revised Cochrane Risk of Bias tool (RoB 2) [16]. Five domains were evaluated: bias due to randomization process, deviations from intended interventions, missing outcome data, bias in outcome measurements, and bias in selection of reported results. Risk of bias assessments were independently performed by two reviewers.

### 2.6. Grading of Certainty of Evidence

Certainty of evidence was assessed using the Grading of Recommendations Assessment, Development, and Evaluation (GRADE) approach for ACD, ICD, ABI, ankle pressure, and adverse events [17]. Certainty of evidence was evaluated across domains of risk of bias, inconsistency, indirectness, imprecision, and publication bias, and was classified as high, moderate, low, or very low. GRADE assessments were performed using GRADEpro GDT (McMaster University, Hamilton, ON, Canada; accessed 7 July 2026).

### 2.7. Statistical Analysis

We conducted a meta-analysis to compare outcomes between the GBE and placebo groups. A total of 13 studies used treadmill protocols for ACD and ICD, whereas 1 study [18] assessed walking distance during flat floor walking at two steps per second. Because treadmill protocols including speed and inclination, varied across trials, walking capacity was converted to estimated caloric expenditure to facilitate comparisons across studies. This method was used in a previously published Cochrane review [13] and was applied consistently across all trials included in the meta-analysis including a recent randomized trial [15]. Accordingly, individual walking-distance data were converted to caloric expenditure (kcal) using treadmill speed, grade, and body weight. The standardized ACD and ICD values were pooled as mean differences (MDs) with 95% confidence intervals (CIs). ACD and ICD were additionally analyzed according to treatment duration (12–16 and 24 weeks). ABI and ankle pressure were synthesized as standardized mean differences with 95% confidence intervals (CIs). Heterogeneity was assessed using I^2^ statistic and Cochran’s Q test, with *p* < 0.10 indicating statistical significance. I^2^ was interpreted in accordance with the Cochrane Handbook [19]. A random-effects model using the DerSimonian–Laird method was applied to account for between-study heterogeneity [20]. A random-effects meta-regression was performed to assess dose-response relationships for ACD. Publication bias for the primary outcome was evaluated using funnel plots, and asymmetry was assessed using Egger’s regression test [21,22].

Sensitivity analyses were planned by excluding studies deemed to have an overall high risk of bias, and also by separately excluding studies with a markedly discrepant effect estimate to assess its influence on pooled effect estimate and heterogeneity. All analyses were performed using R version 4.5.3 (R Foundation for Statistical Computing, Vienna, Austria) with a meta package (version 8.5-0).

## 3. Results

### 3.1. Study Selection and Characteristics

In total, 232 records were identified through database (*n* = 228) and manual searches of the reference lists of the included studies (*n* = 4). Of these, 186 records were screened by title and abstract, and 130 records were further assessed for eligibility. Following full-text assessment, studies were excluded owing to differences in study design, population, intervention duration, intervention outcomes, or control group. Finally, 14 RCTs were included in the meta-analysis (Figure 1).

Table 1 summarizes the characteristics of the 14 studies that used GBE compared with placebo [15,18,23,24,25,26,27,28,29,30,31,32,33,34]. All the included studies enrolled adults with IC due to PAD corresponding to Fontaine stage II. All studies were double-blind RCTs including 10 published and 4 unpublished studies. Other 3 unpublished studies [27,30,32] were identified through published meta-analyses and 1 internal report containing unpublished data [15] was obtained from the manufacturer. The studies included in the review were reported between 1980 and 2026 and were conducted in Germany (*n* = 8), France (*n* = 1), United Kingdom (*n* = 1), Denmark (*n* = 1), Australia (*n* = 1), the United States (*n* = 1), and South Korea (*n* = 1). The treatment duration ranged from 6 to 24 weeks, and the daily GBE dose ranged from 120 to 300 mg/day. A total of 893 participants were randomized across the 14 studies. ACD was reported in 12 studies, ICD in all 14 studies, ABI or ankle pressure in 7 studies, and adverse events in 11 studies.

### 3.2. Risk of Bias

**Figure 2:**
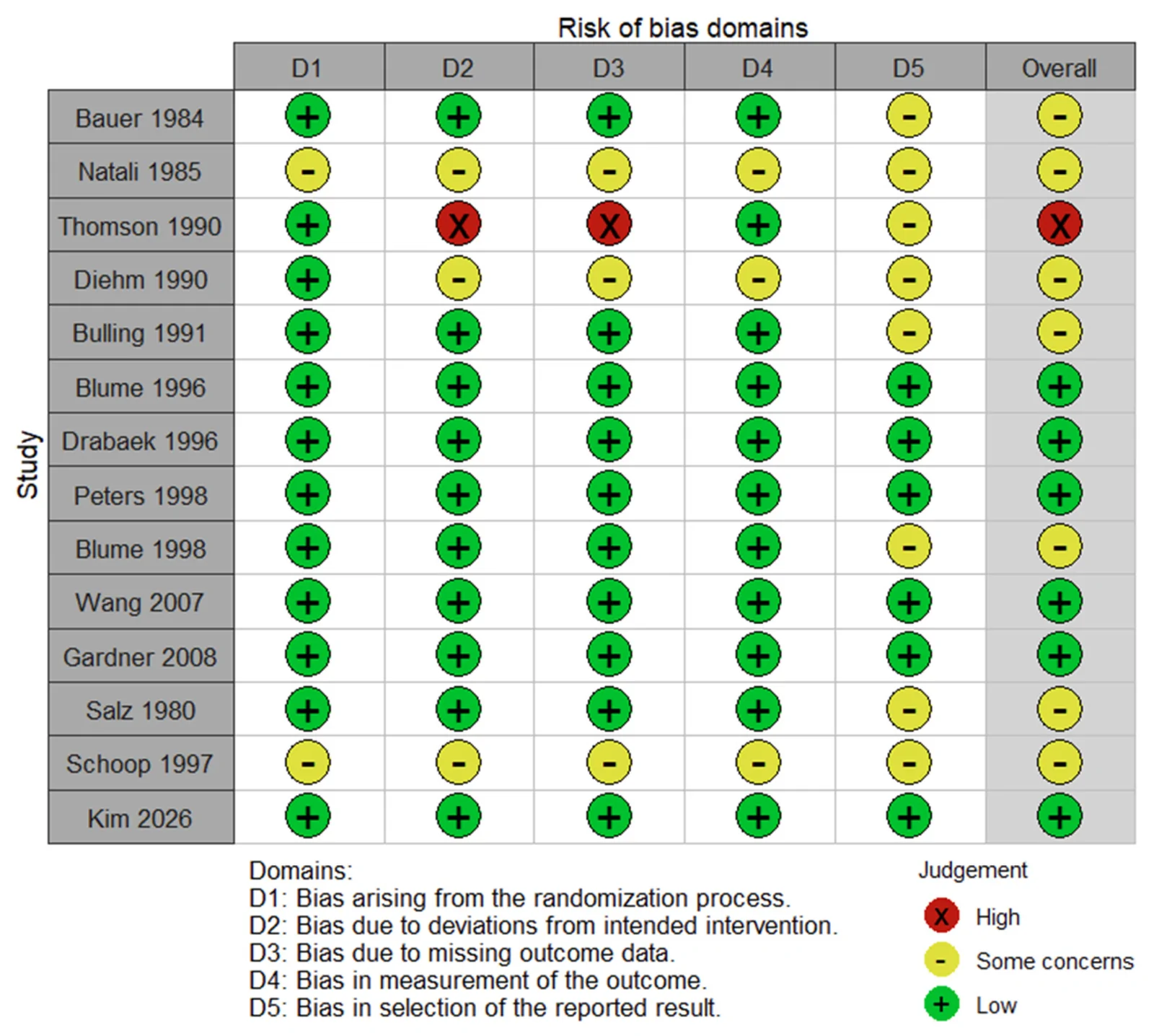
Risk of Bias assessment [15,18,23,24,25,26,27,28,29,30,31,32,33,34].

In the risk of bias assessment, 12 of the 14 studies were deemed to have a low bias risk owing to the randomization process, and 2 studies [30,32] had insufficient information on the randomization sequence generation or allocation concealment. For deviations from the intended interventions and missing outcome data, 10 studies were rated as low risk, whereas three studies [27,30,32] were rated as having some concerns because the available reports provided insufficient information to fully assess these domains. One study [33] was deemed to be high risk in both domains because of its high dropout rate. Bias in outcome measurement was generally low with 11 studies rated as low risk, and the remaining 3 studies [27,30,32] for which only limited information from internal reports was available, were rated to have some concerns [16]. Concerns were most frequent in the selection of reported results; 8 studies [18,23,25,26,27,30,32,33] were rated as having some concerns primarily because pre-specified protocols or statistical analysis plans could not be established. Overall, 6 studies [15,24,28,29,31,34] showed a low risk of bias, 7 studies [18,23,25,26,27,30,32] showed some concerns, and one study [33] was rated as having a high risk of bias (Figure 2).

**Table 1:** Characteristics of included studies.

Author	Year	Study Design	Number Randomized (T/C)	Country	Sex (Male/ Female)	Age, Mean (T/C)	Treatment	Control	Dose	Duration (Weeks)	Outcomes	Treadmill Protocol (Grade, Speed)	Baseline ICD (m), Mean (T/C)	Baseline ACD (m), Mean (T/C)
Bauer [23]	1984	RCT, double-blind	79 (44/35)	Germany	61/18	60.9	*Ginkgo biloba* extract	Placebo	120 mg/day	24	ACD, ICD, ankle pressure, AE	10%, 3 km/h	59/81	111/147
Natali [30]	1985	RCT, double-blind	18 (9/9) ^§^	France	15/3	60.8/66.2	*Ginkgo biloba* extract	Placebo	160 mg/day	24	ACD, ICD, AE	5%, 3 km/h	NR	NR
Thomson [33]	1990	RCT, double-blind	49 (25/24) ^§^	United Kingdom	Not reported	Not reported	*Ginkgo biloba* extract	Placebo	Not specified	24	ICD, ankle pressure, AE	10°, 4 km/h	80.7/100.6	NA
Diehm [27]	1990	RCT, multicenter, double-blind, pilot	40 (22/18) ^§^	Germany	38/2	62.2/63.0	*Ginkgo biloba* extract	Placebo	120 mg/day	12	ACD, ICD, AE	10%, 5 km/h	NR	NR
Bulling [26]	1991	RCT, double-blind	36 (17/16) ^‡^	Germany	24/9	62.8/63.3	*Ginkgo biloba* extract	Placebo	160 mg/day	24	ACD, ICD, ankle pressure, AE	10%, 3 km/h	85/78	125/165
Blume [24]	1996	RCT, double-blind	60 (30/30)	Germany	42/18	71/68.6 *	*Ginkgo biloba* extract	Placebo	120 mg/day	24	ACD, ICD, ABI, AE	12%, 3 km/h	96/111.5 *	126/143.5 *
Drabaek [28]	1996	RCT, double-blind, crossover	20 (9/9) ^‡,§^	Denmark	11/9	74 *	*Ginkgo biloba* extract	Placebo	120 mg/day	12	ACD, ICD, ABI, AE	8–16°, 2.5–4.0 km/h (set per patient)	92 *	188 *
Peters [31]	1998	RCT, multicenter, double-blind	111 (53/58)	Germany	63/46	62.8/61.2	*Ginkgo biloba* extract	Placebo	120 mg/day	24	ACD, ICD, ABI, AE	12%, 3 km/h	108.5/105.2	NR
Blume [25]	1998	RCT, double-blind	41 (21/20)	Germany	29/11	66.4/68.2	*Ginkgo biloba* extract	Placebo	160 mg/day	24	ACD, ICD, ABI, AE	12%, 3 km/h	81.4/74.0	97.9/92.8
Wang [34]	2007	RCT, double-blind	22 (11/11)	Australia	20/2	71.6/71.2	*Ginkgo biloba* extract	Placebo	240 mg/day	12	ACD, ICD, ABI	0% increasing by 2% every 2 min, 3.2 km/h	96/114	204/300
Gardner [29]	2008	RCT, double-blind	62 (31/31)	United States	37/25	70/69	*Ginkgo biloba* extract	Placebo	300 mg/day	16	ACD, ICD, ABI	10%, 3.2 km/h	60/59	201/183
Salz [18]	1980	RCT, double-blind, crossover	29 (13/13) ^‡,§^	Germany	11/18	69.0/64.4 ^†^	*Ginkgo biloba* extract	Placebo	160 mg/day	6	ACD, ICD	Floor walking, 2 steps/s	NR	NR
Schoop [32]	1997	RCT, double-blind	205 (105/100) ^§^	Germany	157/48	64.3/67.2	*Ginkgo biloba* extract	Placebo	120 mg/day	24	ICD, AE	12%, 3 km/h	NR	NA
Kim [15]	2026	RCT, multicenter, double-blind	121 (60/61)	South Korea	112/9	68.0/66.7	*Ginkgo biloba* extract	Placebo	160 mg/day	24	ACD, ICD, ABI, AE	12%, 3.2 km/h	194.5/189.6	352.8/354.7

RCT, Randomized Controlled Trial; T, Treatment group (*Ginkgo biloba* extract); C, Control group (placebo); ACD, Absolute Claudication Distance; ICD, Initial Claudication Distance; ABI, Ankle-Brachial Index; AE, Adverse Events; NA, Not Available; NR, Not Reported. * Median values are reported. ^†^ Age values are mean ages by sex (male/female) not by treatment/control group. ^‡^ Total is the number randomized; T/C split reflects the number evaluated after dropouts. ^§^ T/C split as reported in Cochrane review; the original publication was either unpublished/inaccessible or did not report the group-level (T/C) allocation.

### 3.3. Effects of Ginkgo biloba Extract

#### 3.3.1. Absolute Claudication Distance

**Figure 3:**
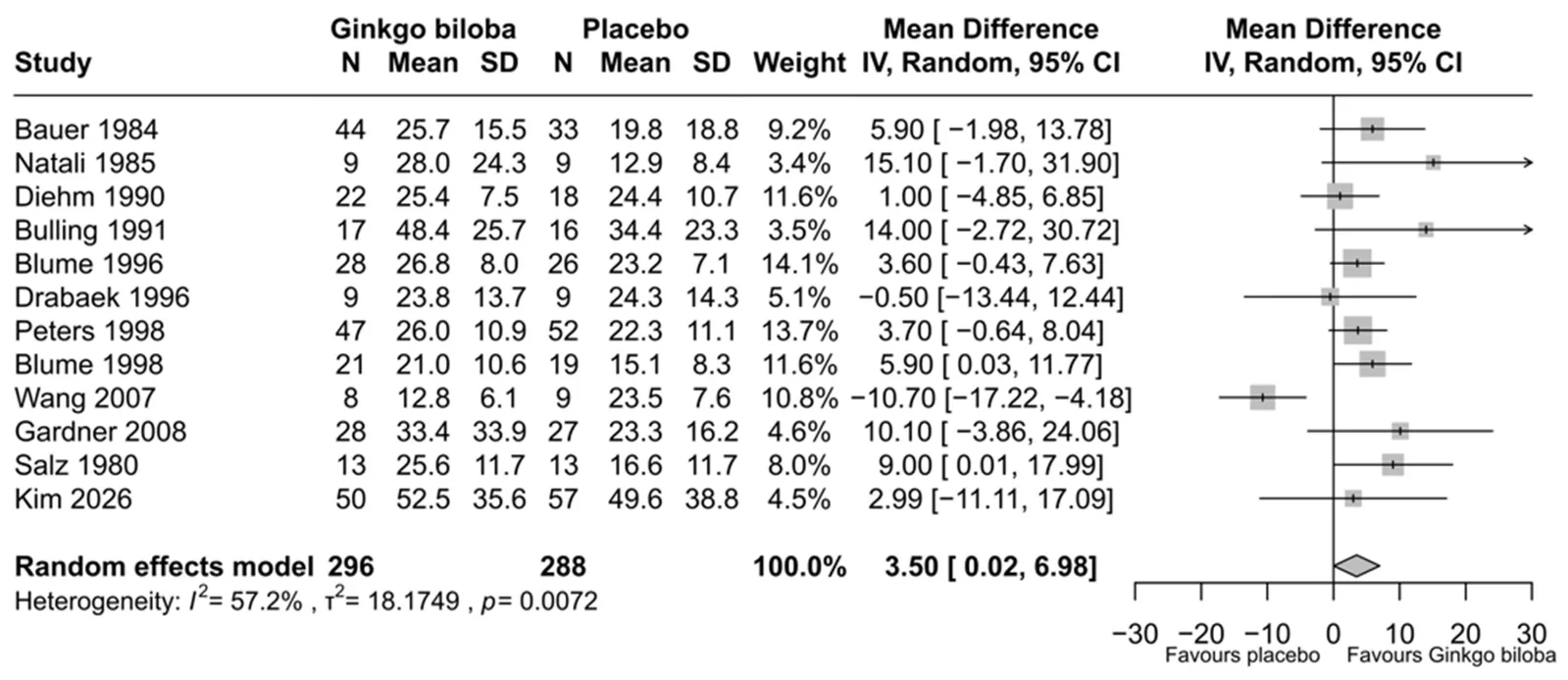
Forest plot of absolute claudication distance (expressed as kilocalories) at the end of the study: *Ginkgo biloba* versus placebo [15,18,23,24,25,26,27,28,29,30,31,34]. SD, Standard Deviation; CI, Confidence Interval; IV, Inverse Variance.

At the end of the study, 12 studies [15,18,23,24,25,26,27,28,29,30,31,34] comprising 296 participants in the *Ginkgo biloba* group and 288 participants in the placebo group were included in the ACD analysis. The overall effect size was 3.50 kcal (95% CI, 0.02 to 6.98; *p* = 0.049) for the *Ginkgo biloba* group (Figure 3). 

**Figure 4:**
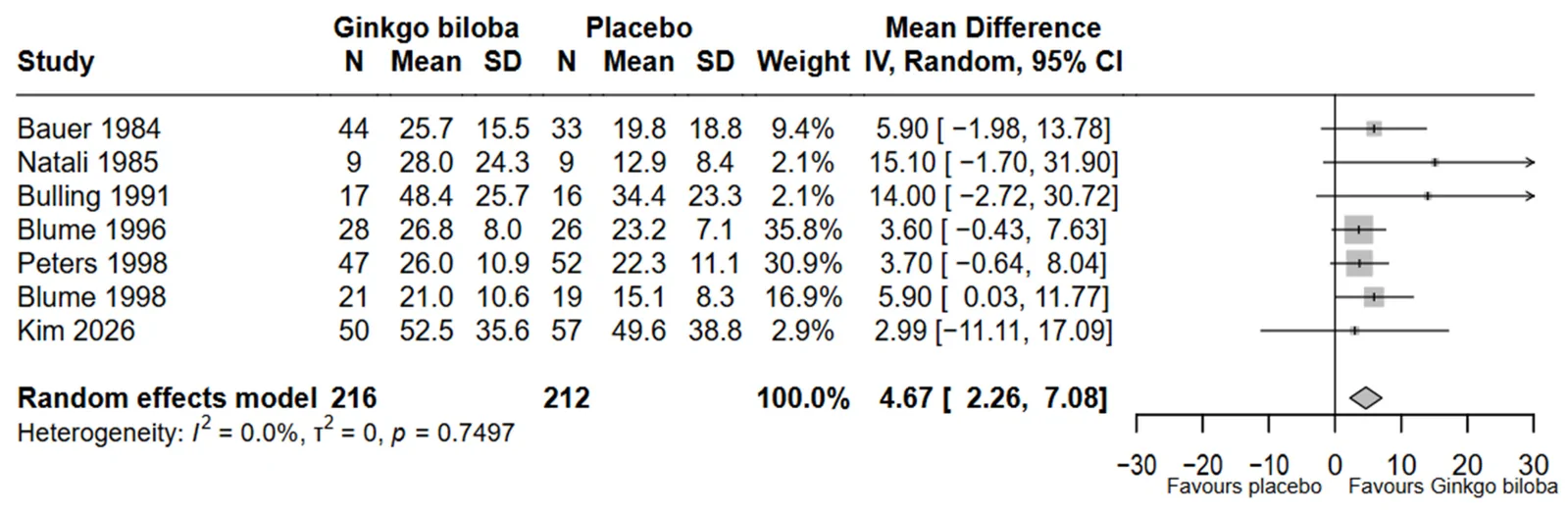
Forest plot of absolute claudication distance (expressed as kilocalories) at 24 weeks: *Ginkgo biloba* versus placebo [15,23,24,25,26,30,31]. SD, Standard Deviation; CI, Confidence Interval; IV, Inverse Variance.

The effect size was calculated according to treatment duration. At 24 weeks, ACD data from 7 studies [15,23,24,25,26,30,31] comprising 216 participants administered *Ginkgo biloba* and 212 administered placebo were available. The overall effect size was 4.67 kcal (95% CI, 2.26 to 7.08; *p* < 0.001) for the *Ginkgo biloba* group (Figure 4). 

**Figure 5:**
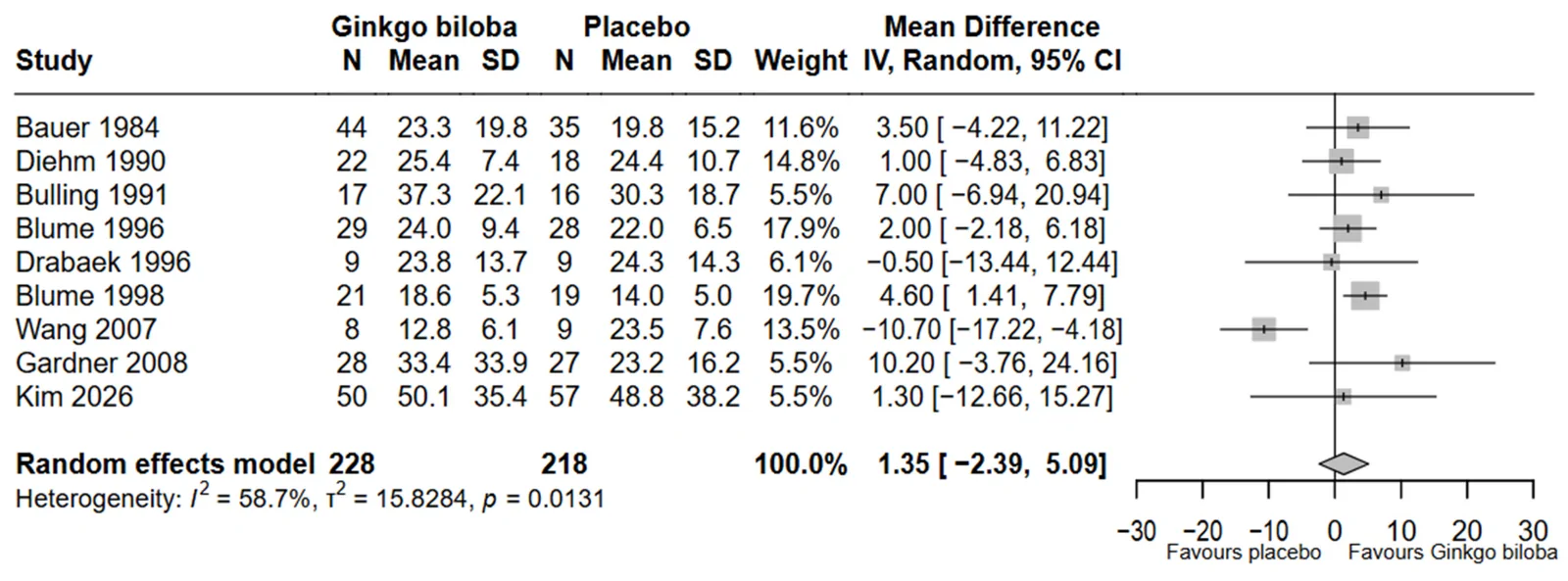
Forest plot of absolute claudication distance (expressed as kilocalories) at 12–16 weeks: *Ginkgo biloba* versus placebo [15,23,24,25,26,27,28,29,34]. SD, Standard Deviation; CI, Confidence Interval; IV, Inverse Variance.

At 12–16 weeks, data from 9 studies [15,23,24,25,26,27,28,29,34] showed an overall effect size of 1.35 kcal (95% CI, −2.39 to 5.09; *p* = 0.48) (Figure 5).

#### 3.3.2. Initial Claudication Distance

At the end of the study, all 14 studies [15,18,23,24,25,26,27,28,29,30,31,32,33,34] comprising 423 participants in the *Ginkgo biloba* group and 407 participants in the placebo group were included in the ICD analysis. The overall effect size was 1.89 kcal (95% CI, −0.80 to 4.58; *p* = 0.169) (Supplementary Figure S1). At 24 weeks, data from 9 studies [15,23,24,25,26,30,31,32,33] showed an overall effect size of 2.20 kcal (95% CI, −1.81 to 6.21; *p* = 0.28) between the *Ginkgo biloba* and placebo groups (Supplementary Figure S2). At 12–16 weeks, data from 10 studies [15,23,24,25,26,27,28,29,31,34] showed an overall effect size of 1.50 kcal (95% CI, −0.00 to 3.01; *p* = 0.050) (Supplementary Figure S3). 

#### 3.3.3. Ankle-Brachial Index and Ankle Pressure

A total of 7 studies [15,23,26,28,29,33,34] contributed eight datasets to the quantitative analysis of ABI and ankle pressure, and 1 study provided separate data for dorsalis pedis and posterior tibial arteries. These datasets comprised 194 participants in the *Ginkgo biloba* group and 187 participants in the placebo group. Standardized mean differences were used to pool ABI and ankle pressure. The overall effect size was −0.17 (95% CI, −0.47 to 0.12; *p* = 0.24) between the *Ginkgo biloba* and placebo groups at the end of the study (Supplementary Figure S4).

#### 3.3.4. Adverse Events

A total of 11 studies reported adverse events and safety findings [15,23,24,25,26,27,28,30,31,32,33]. Safety outcomes were synthesized descriptively because the definitions, classifications, and reporting methods of adverse events varied substantially across trials (Supplementary Table S4). Four studies reported no adverse events or side effects [25,26,27,32]. In two further studies, events occurred only in placebo-treated participants [31,33]. Events in the *Ginkgo biloba* group were stomach pain and nausea (one event each) [24] and blood in urine in one participant with bladder cancer, which the investigators considered possibly drug-related [23]. Another study reported adverse reactions without specifying their type [30]. In one study, two participants discontinued because of severe dyspnea and a transient cerebral ischemic attack, which was attributed to withdrawal of acetylsalicylic acid [28]. In the most recent study [15], the overall incidence of adverse events was similar between the GNX80 (GBE formulation, 80 mg twice daily, 160 mg/day) and placebo groups (35.1% vs. 26.2%; *p* = 0.30). Serious adverse events were not frequent and no adverse drug reactions were reported in the GNX80 group. Overall, no new or unexpected safety signals were identified across the studies.

#### 3.3.5. Dose–Response Relationship

The daily doses of *Ginkgo biloba* varied across studies included in the primary outcome analysis. Among the 12 studies with reported dosage information, 5 studies [23,24,27,28,31] evaluated 120 mg/day, 5 studies [15,18,25,26,30] evaluated 160 mg/day, 1 study [34] evaluated 240 mg/day, and 1 study [29] evaluated 300 mg/day. Meta-regression across 12 studies showed no significant association between daily dose of *Ginkgo biloba* and treatment effect on ACD (regression coefficient β = −0.31; 95% CI, −1.00 to 0.38; *p* = 0.37, R^2^ = 12.7%; residual I^2^ = 53.3%). 

### 3.4. Publication Bias and Sensitivity Analysis

Funnel plots were constructed for ACD analyses at the end of the study and at 24 weeks (Supplementary Figure S5). Egger’s regression test showed no evidence of funnel plot asymmetry in the end-of-study analysis (*p* = 0.41). Egger’s test was not performed for the 24-week subgroup because it included only seven studies, fewer than the 10 recommended for reliable assessment of funnel plot asymmetry [21,22]. An influence analysis was then performed after excluding a study with a markedly discrepant effect estimate, resulting in 11 studies [15,18,23,24,25,26,27,28,29,30,31]. The pooled effect remained significant (MD, 4.42 kcal; 95% CI, 2.31 to 6.53) with no observed heterogeneity (I^2^ = 0.0%; *p* = 0.73). Sensitivity analysis excluding the study at a high risk of bias [33] was not performed because ACD was not reported. Therefore, a sensitivity analysis excluding studies at high risk of bias was not applicable. In a post hoc sensitivity analysis of the 24-week ACD outcome excluding one large trial [15], the pooled effect remained significant (MD, 4.72 kcal; 95% CI, 2.27 to 7.17; *p* < 0.001; I^2^ = 0.0%) and was consistent with the primary 24-week estimate (MD, 4.67 kcal; 95% CI, 2.26 to 7.08).

### 3.5. GRADE Assessment

Using the GRADE approach, certainty of evidence was rated as moderate for ACD, ABI, ankle pressure, and adverse events, and low for ICD. The certainty of evidence for ACD was downgraded by one level due to inconsistency (I^2^ = 57.2%). ICD was downgraded for inconsistency and imprecision, and ABI or ankle pressure and adverse events were downgraded for imprecision (Supplementary Table S6).

## 4. Discussion

GBE has been investigated as a potential treatment for IC based on its proposed vasoactive and antioxidant properties including modulation of peripheral blood flow and inhibition of platelet aggregation [12]. In this updated systematic review and meta-analysis of 14 randomized controlled trials, GBE was associated with a statistically significant but modest improvement in ACD compared with placebo (3.50 kcal; 95% CI, 0.02 to 6.98). The pooled effects of 3.50 kcal (at the end of the study) and 4.67 kcal (24 weeks) correspond to approximately 60 m and 81 m, respectively, for a 70 kg patient walking on a flat treadmill at an average speed of 3.2 km/h, which is most comparable with walking in daily life. However, the exploratory subgroup analysis suggested the treatment effects varied by follow-up duration, with a greater effect observed at 24 weeks than at 12–16 weeks. Findings for ICD were less conclusive. The pooled estimate numerically favored GBE but this difference was not statistically significant, and the certainty of evidence was low. No improvement was observed in ABI or ankle pressure. This is biologically plausible, as ABI primarily reflects proximal macrovascular obstruction and may be relatively insensitive to functional improvements that occur without structural changes in arterial stenosis, whereas walking performance may be influenced by microcirculatory adaptation, skeletal muscle metabolism, endothelial function, and exercise tolerance. Consistent with this interpretation, exercise therapy improves pain-free and maximal walking distance without a significant change in ABI [35]. In the included trials, GBE was generally well tolerated with an overall incidence of adverse events comparable to that of placebo, supported by moderate certainty of evidence (Supplementary Table S6). Overall, these findings suggest that GBE treatment is associated with a modest improvement in walking performance, particularly after extended exposure (24 weeks), but do not establish consistent effects on exertional pain onset, resting limb perfusion, or dose–response relationships.

The findings of this study provided a meaningful update to a previous Cochrane systematic review published in 2013 [13]. The 2013 Cochrane review reported a pooled effect of 3.57 kcal (95% CI, −0.1 to 7.23; I^2^ = 61%) that did not reach statistical significance when all trial durations were analyzed together. However, subgroup analysis in the Cochrane review indicated that trials with a 24-week follow-up period (six trials) showed significantly greater ACD in the GBE group than in the placebo group. 

The present analysis alters this landscape primarily through the incorporation of more recent data, most notably one large phase 4 multicenter trial [15]. This randomized, double-blind, placebo-controlled trial evaluated high-dose GBE (GNX80; 80 mg twice daily, 160 mg/day) versus placebo over a 24-week period using a standardized treadmill testing protocol (12% grade, 3.2 km/h) in patients with stable Fontaine stage IIb IC across 15 university hospitals in South Korea. In this trial, GBE treatment resulted in a statistically significant improvement in maximal walking distance (MWD) compared with placebo (change from baseline: 69.8 ± 136.5 m vs. 19.3 ± 126.8 m; *p* = 0.041).

These findings are consistent with those of several landmark 24-week clinical trials. Blume et al. [24] reported that in 60 patients with Fontaine stage IIb IC who had undergone at least 6 months of physical training without further improvement, 24 weeks of EGb 761 (120 mg/day) led to a significant increase in MWD (mean gain: 50.5 m vs. 15.5 m in placebo; *p* < 0.001) along with a substantial increase in pain-free walking distance (PFWD; mean gain: 41.0 m vs. 8.0 m). Similarly, Peters et al. [31] demonstrated sustained improvements in PFWD across 8, 16, and 24 weeks (*p* < 0.05), and a significant prolongation of MWD with EGb 761 (120 mg/day) at 24 weeks (*p* = 0.026). Furthermore, early randomized double-blind trials [18,23] also documented significant increase in total walking distance after 6 months of standardized GBE treatment (120–160 mg/day) compared with placebo (*p* < 0.001).

With the addition of Kim et al. [15], the effect on ACD reached statistical significance, representing an almost identical point estimate to that reported by the 2013 Cochrane review. However, heterogeneity of findings observed in the present study was similar to that of the 2013 Cochrane review (I^2^ = 57.2% vs. 61%), indicating that the underlying inconsistencies across trials could not be fully resolved by this update. Rather, the shift to statistical significance most plausibly reflects improved precision and statistical power from a larger combined sample, together with the near-identical point estimate across both syntheses, suggesting a consistent underlying signal rather than a newly emergent effect. Sensitivity analyses in the present review showed that excluding studies with a high risk of bias was not applicable because the only study which was deemed to have a high risk of bias did not report ACD and therefore was not included in the primary ACD analysis. In contrast, the exclusion of a single influential outlier trial [34] substantially strengthened the pooled effect (Supplementary Table S5). Notably, this was the only trial with an effect estimate favoring placebo and differed from the other trials in that GBE was administered alongside a structured exercise program.

An important finding of the present analysis is that improvements in ACD were observed in trials evaluating 24 weeks of treatment, whereas meta-regression showed no significant association between daily dose and treatment effect. Although these findings should be interpreted cautiously, this pattern may be explained by the proposed pharmacological properties of GBE. GBE has been reported to exert antioxidant, vasodilatory, and metabolic regulatory effects that may contribute to vascular protection and improved peripheral circulation [9]. Given that functional impairment in patients with IC involves not only macrovascular obstruction but also microcirculatory dysfunction and altered skeletal muscle metabolism, the cumulative effects of these pharmacological actions may contribute to gradual improvements in exercise tolerance and walking performance with sustained treatment. This provides a plausible biological explanation for the more consistent improvements in ACD observed after 24 weeks of treatment, despite the absence of a significant dose–response relationship.

### 4.1. Clinical Implications

These findings should be interpreted within the broader clinical context of IC management, where cilostazol is an established pharmacological treatment, and additional therapeutic options remain valuable. Although cilostazol has been reported to increase absolute claudication distance by approximately 40 to 100 m over placebo, our pooled effect (approximately 60 m overall and 81 m at 24 weeks) indicates an improvement in walking performance within the range reported for cilostazol. However, direct comparisons should be interpreted cautiously because of differences in study populations, treadmill protocols, and outcome assessments. GBE demonstrated an adverse event profile comparable to placebo in the included trials, consistent with previous reports of favorable tolerability [36]. Taken together, these findings suggest that GBE represents a viable alternative or adjunctive therapeutic option for patients with IC, offering a balance of functional benefit and tolerability.

In clinical practice, improvements in walking performance appeared greater at the 24-week assessment than at earlier time points. Utilizing standardized walking assessments and identifying patient characteristics associated with the treatment response such as disease severity, concomitant medications, and baseline walking capacity, can further maximize the therapeutic efficacy of GBE in routine care.

### 4.2. Strengths and Limitations

This review had several strengths. This review represented updated evidence on GBE for IC over a decade incorporating a substantially larger and more contemporary body of trial data than the 2013 Cochrane review and included a large multicenter trial which was not evaluated in previous systematic reviews. Notably, individual participant-level data were obtained directly from investigators for this large multicenter study, allowing it to be incorporated with the same rigor as published trials rather than relying on secondary or incomplete reporting. The use of a standardized kilocalorie-based outcome, consistent with the methodology of the 2013 Cochrane review, allowed for a direct comparison across syntheses despite variations in treadmill testing protocols.

Despite these strengths, several limitations of this study must be acknowledged. First, heterogeneity remained substantial for ACD which was likely due to a wide range of GBE doses (120–300 mg/day), sample sizes (18–205 participants), and study designs across trials conducted in seven countries over more than four decades. However, subgroup analyses by treatment duration and meta-regression by dose provided insights into the potential sources of this variability. Second, the certainty of evidence was rated low for ICD and moderate for hemodynamic indices and adverse events indicating inconsistency and imprecision of these outcomes. Therefore, firm conclusions regarding these secondary outcomes cannot be drawn from the current evidence. Third, all included studies enrolled patients with intermittent claudication corresponding to Fontaine stage II disease. Therefore, the present findings are applicable only to this patient population and should not be extrapolated to patients with asymptomatic PAD or advanced disease stages, including chronic limb-threatening ischemia. Fourth, six of the 14 trials were at low risk of bias, seven raised some concerns, and one was at high risk but did not report ACD. Concerns mainly related to selective reporting in older trials and limited information in unpublished reports. These limitations should be considered when interpreting the findings. Fifth, we included unpublished studies to reduce the risk of publication bias. However, some of these studies have not undergone peer review. They were also included in a previous meta-analysis. Finally, this study received partial industry funding, and one author was affiliated with a contract research organization. Although the sponsor had no role in the study, we note these relationships so that readers can consider them when interpreting the findings. 

## 5. Conclusions

In this updated systematic review and meta-analysis, GBE was associated with a statistically significant but modest improvement in ACD compared with placebo, with a significant benefit observed in trials evaluating 24 weeks of treatment. Importantly, this improvement in walking performance was achieved with an adverse event profile comparable to that of placebo, supporting the safety and tolerability of GBE in clinical practice. Given the constrained pharmacological landscape and the tolerability challenges of existing agents, these findings suggest that GBE may provide a modest improvement in walking capacity and may represent a useful, well-tolerated therapeutic option for patients with intermittent claudication secondary to peripheral artery disease, particularly with evidence of benefit in trials evaluating 24 weeks of treatment.

## Data Availability

Data analyzed in this systematic review were obtained from published studies and published meta-analyses cited in the article and its Supplementary Materials. Three unpublished studies were identified through published meta-analyses. For one additional unpublished study, an internal report containing unpublished data was obtained from the manufacturer, and individual participant-level data from this study were made available to the investigators and included in the analyses. These individual participant-level data are not publicly available.

## Supplementary Materials

The following supporting information can be downloaded at: https://www.mdpi.com/article/10.3390/jcm15218606/s1, Table S1: PRISMA 2020 checklist; Table S2: PRISMA 2020 abstract checklist; Table S3: Database search strategies; Table S4: Adverse events; Table S5: Sensitivity analysis; Table S6: GRADE assessment; Figure S1: Forest plot of initial claudication distance (expressed as kilocalories) at the end of the study: *Ginkgo biloba* versus placebo; Figure S2: Forest plot of initial claudication distance (expressed as kilocalories) at 24 weeks: *Ginkgo biloba* versus placebo; Figure S3: Forest plot of initial claudication distance (expressed as kilocalories) at 12–16 weeks: *Ginkgo biloba* versus placebo; Figure S4: Forest plot of ABI & ankle pressure at the end of the study: *Ginkgo biloba* versus placebo; Figure S5: Funnel plots for ACD.
