# Supplementary material for: Ginkgo biloba Extract for Intermittent Claudication in Patients with Peripheral Arterial Disease: A Systematic Review and Meta-Analysis of Randomized Controlled Trials
Source: J Clin Med. 2026 Nov 4;15(21):8606. doi: 10.3390/jcm15218606 (PMC13763640; doi:10.3390/jcm15218606)
Supplement: Supplementary file 1 [file jcm-15-08606-s001.zip › jcm-4570750-supplementary.pdf]

SUPPLEMENTARY MATERIALS

Supplementary Table S1. PRISMA 2020 checklist

Section and Topic	Item #	Checklist item	Location where item is reported
**TITLE**			
Title	1	Identify the report as a systematic review.	Page 1
**ABSTRACT**			
Abstract	2	See the PRISMA 2020 for Abstracts checklist.	Page 1
**INTRODUCTION**			
Rationale	3	Describe the rationale for the review in the context of existing knowledge.	Introduction, paragraphs 1–4
Objectives	4	Provide an explicit statement of the objective(s) or question(s) the review addresses.	Introduction, final paragraph
**METHODS**			
Eligibility criteria	5	Specify the inclusion and exclusion criteria for the review and how studies were grouped for the syntheses.	Section 2.3 (Eligibility Criteria); Section 2.1 (Outcomes); Section 2.7 (grouping for syntheses: 12–16 vs 24 weeks)
Information sources	6	Specify all databases, registers, websites, organisations, reference lists and other sources searched or consulted to identify studies. Specify the date when each source was last searched or consulted.	Section 2.2 (PubMed, EMBASE, Cochrane Library, CINAHL; searched April 24, 2026; reference lists; manufacturers; Cochrane review)
Search strategy	7	Present the full search strategies for all databases, registers and websites, including any filters and limits used.	Section 2.2; Supplementary Table S3 (full strategies)
Selection process	8	Specify the methods used to decide whether a study met the inclusion criteria of the review, including how many reviewers screened each record and each report retrieved, whether they worked independently, and if applicable, details of automation tools used in the process.	Section 2.4; Figure 1
Data collection process	9	Specify the methods used to collect data from reports, including how many reviewers collected data from each report, whether they worked independently, any processes for obtaining or confirming data from study investigators, and if applicable, details of automation tools used in the process.	Section 2.5, paragraph 1; Section 2.2; Data Availability Statement
Data items	10a	List and define all outcomes for which data were sought. Specify whether all results that were compatible with each outcome domain in each study were sought (e.g. for all measures, time points, analyses), and if not, the methods used to decide which results to collect.	Section 2.1
	10b	List and define all other variables for which data were sought (e.g. participant and intervention characteristics, funding sources). Describe any assumptions made about any missing or unclear information.	Section 2.5, paragraph 1; Table 1

Study risk of bias assessment	11	Specify the methods used to assess risk of bias in the included studies, including details of the tool(s) used, how many reviewers assessed each study and whether they worked independently, and if applicable, details of automation tools used in the process.	Section 2.5, paragraph 2
Effect measures	12	Specify for each outcome the effect measure(s) (e.g. risk ratio, mean difference) used in the synthesis or presentation of results.	Section 2.7 (MD in kcal for ACD and ICD; SMD for ABI and ankle pressure); Section 3.3.4 (adverse events)
Synthesis methods	13a	Describe the processes used to decide which studies were eligible for each synthesis (e.g. tabulating the study intervention characteristics and comparing against the planned groups for each synthesis (item #5)).	Section 2.1, 2.3 and 2.7; Table 1
	13b	Describe any methods required to prepare the data for presentation or synthesis, such as handling of missing summary statistics, or data conversions.	Section 2.7; Section 2.5
	13c	Describe any methods used to tabulate or visually display results of individual studies and syntheses.	Table 1; Figures 2–5; Supplementary Figures S1–S5
	13d	Describe any methods used to synthesize results and provide a rationale for the choice(s). If meta-analysis was performed, describe the model(s), method(s) to identify the presence and extent of statistical heterogeneity, and software package(s) used.	Section 2.7
	13e	Describe any methods used to explore possible causes of heterogeneity among study results (e.g. subgroup analysis, meta-regression).	Section 2.7
	13f	Describe any sensitivity analyses conducted to assess robustness of the synthesized results.	Section 2.7
Reporting bias assessment	14	Describe any methods used to assess risk of bias due to missing results in a synthesis (arising from reporting biases).	Section 2.7
Certainty assessment	15	Describe any methods used to assess certainty (or confidence) in the body of evidence for an outcome.	Section 2.6
**RESULTS**			
Study selection	16a	Describe the results of the search and selection process, from the number of records identified in the search to the number of studies included in the review, ideally using a flow diagram.	Section 3.1; Figure 1
	16b	Cite studies that might appear to meet the inclusion criteria, but which were excluded, and explain why they were excluded.	Figure 1 (exclusion reasons by category); Section 3.1
Study characteristics	17	Cite each included study and present its characteristics.	Table 1; Section 3.1
Risk of bias in studies	18	Present assessments of risk of bias for each included study.	Section 3.2; Figure 2
Results of individual studies	19	For all outcomes, present, for each study: (a) summary statistics for each group (where appropriate) and (b) an effect estimate and its precision (e.g. confidence/credible interval), ideally using structured tables or plots.	Figures 3–5; Supplementary Figures S1–S4; Supplementary Table S4
Results of syntheses	20a	For each synthesis, briefly summarise the characteristics and risk of bias among contributing studies.	Sections 3.1 and 3.2; Section 3.3

	20b	Present results of all statistical syntheses conducted. If meta-analysis was done, present for each the summary estimate and its precision (e.g. confidence/credible interval) and measures of statistical heterogeneity. If comparing groups, describe the direction of the effect.	Sections 3.3.1–3.3.4; Figures 3–5
	20c	Present results of all investigations of possible causes of heterogeneity among study results.	Section 3.3.1 (duration); Section 3.3.5 (dose)
	20d	Present results of all sensitivity analyses conducted to assess the robustness of the synthesized results.	Section 3.4; Supplementary Table S5
Reporting biases	21	Present assessments of risk of bias due to missing results (arising from reporting biases) for each synthesis assessed.	Section 3.4; Supplementary Figure S5
Certainty of evidence	22	Present assessments of certainty (or confidence) in the body of evidence for each outcome assessed.	Section 3.5; Supplementary Table S6
**DISCUSSION**			
Discussion	23a	Provide a general interpretation of the results in the context of other evidence.	Section 4 Discussion, paragraphs 1–6
	23b	Discuss any limitations of the evidence included in the review.	Strengths and limitations, paragraph 2
	23c	Discuss any limitations of the review processes used.	Strengths and limitations, paragraph 2
	23d	Discuss implications of the results for practice, policy, and future research.	Clinical implications section
**OTHER INFORMATION**			
Registration and protocol	24a	Provide registration information for the review, including register name and registration number, or state that the review was not registered.	Section 2, paragraph 1
	24b	Indicate where the review protocol can be accessed, or state that a protocol was not prepared.	Section 2, paragraph 1
	24c	Describe and explain any amendments to information provided at registration or in the protocol.	Section 3.4
Support	25	Describe sources of financial or non-financial support for the review, and the role of the funders or sponsors in the review.	Funding statement
Competing interests	26	Declare any competing interests of review authors.	Conflicts of Interest statement; author affiliations
Availability of data, code and other materials	27	Report which of the following are publicly available and where they can be found: template data collection forms; data extracted from included studies; data used for all analyses; analytic code; any other materials used in the review.	Data Availability Statement; Supplementary Tables S1–S6 and Figures S1–S5

Supplementary Table S2. PRISMA 2020 abstract checklist

Section and Topic	Item #	Checklist item	Reported (Yes/No)
**TITLE**			
Title	1	Identify the report as a systematic review.	Yes
**BACKGROUND**			
Objectives	2	Provide an explicit statement of the main objective(s) or question(s) the review addresses.	Yes
**METHODS**			
Eligibility criteria	3	Specify the inclusion and exclusion criteria for the review.	Yes
Information sources	4	Specify the information sources (e.g. databases, registers) used to identify studies and the date when each was last searched.	Yes
Risk of bias	5	Specify the methods used to assess risk of bias in the included studies.	Yes
Synthesis of results	6	Specify the methods used to present and synthesise results.	Yes
**RESULTS**			
Included studies	7	Give the total number of included studies and participants and summarise relevant characteristics of studies.	Yes
Synthesis of results	8	Present results for main outcomes, preferably indicating the number of included studies and participants for each. If meta-analysis was done, report the summary estimate and confidence/credible interval. If comparing groups, indicate the direction of the effect (i.e. which group is favoured).	Yes
**DISCUSSION**			
Limitations of evidence	9	Provide a brief summary of the limitations of the evidence included in the review (e.g. study risk of bias, inconsistency and imprecision).	Yes
Interpretation	10	Provide a general interpretation of the results and important implications.	Yes
**OTHER**			
Funding	11	Specify the primary source of funding for the review.	No (Funding not reported in the abstract)
Registration	12	Provide the register name and registration number.	No (PROSPERO registration (CRD420261424296) is reported in Methods, not in abstract.)

Table S3. Database search strategies

Database	No.	Search Query
**PubMed**	#1	"Intermittent Claudication"[Mesh]
	#2	"Intermittent Claudication"[TW] OR "intermittent arterial claudication"[TW] OR "intermittent claudicatio"[TW] OR "intermittent claudications"[TW] OR "intermittent vascular claudication"[TW] OR "leg ischaemia pain"[TW] OR "leg ischemia pain"[TW] OR "claudicant"[TW] OR "claudicants"[TW]
	#3	"Peripheral Arterial Disease"[Mesh]
	#4	"Peripheral Arterial Disease"[TW] OR "Peripheral Arterial Diseases"[TW] OR "Peripheral Artery Disease"[TW] OR "Peripheral Artery Diseases"[TW] OR "arterial obliterative disease"[TW] OR "arterial occlusive diseases"[TW] OR "artery chronic occlusive disease"[TW] OR "artery obliterative disease"[TW] OR "artery occlusive disease"[TW] OR "chronic arterial occlusion disease"[TW] OR "chronic artery obstruction"[TW] OR "chronic artery occlusion"[TW] OR "obliterative arterial disease"[TW] OR "obliterative artery disease"[TW] OR "obstructive arterial disease"[TW] OR "obstructive artery disease"[TW] OR "occlusive arterial disease"[TW] OR "occlusive artery disease"[TW] OR "PAD"[TW] OR "PAOD"[TW] OR "peripheral arterial disorder"[TW] OR "peripheral arterial obstructive disease"[TW] OR "peripheral artery obstructive disease"[TW] OR "peripheral atherosclerosis"[TW] OR "peripheral obliterative arterial disease"[TW] OR "peripheral obliterative vascular disease"[TW] OR "peripheral obstructive artery disease"[TW] OR "peripheral occlusive arterial disease"[TW] OR "peripheral occlusive artery disease"[TW] OR "peripheral occlusive disease"[TW] OR "peripheral vascular occlusive disease"[TW]
	#5	"Peripheral Arterial Occlusive Disease 1"[Supplementary Concept]
	#6	"peripheral arterial occlusive disease"[TW] OR "peripheral arterial occlusive diseases"[TW] OR "Fontaine stage II"[TW] OR "Rutherford stage"[TW]
	**#7**	**#1 OR #2 OR #3 OR #4 OR #5 OR #6**
	#8	"Ginkgo biloba"[Mesh]
	#9	"Ginkgo biloba"[TW] OR "Ginkgo bilobas"[TW] OR "Gingko"[TW] OR "Gingkos"[TW] OR "Gingko biloba"[TW] OR "Gingko bilobas"[TW] OR "Ginkgo"[TW] OR "Ginkgos"[TW] OR "Ginko"[TW] OR "Ginkos"[TW]
	#10	"Ginkgo Extract"[Mesh]
	#11	"Ginkgo biloba extract"[Supplementary Concept]
	#12	"Ginkgo leaf extract"[TW] OR "Tebofortran"[TW] OR "Tebokan"[TW] OR "Tebonin"[TW] OR "EGb-761"[TW] OR "EGb761"[TW] OR "GBE 761 ONC"[TW] OR "Rokan"[TW] OR "Tanakan"[TW] OR "GBE-761"[TW] OR "Ginkgo biloba extract 761"[TW] OR "gingko biloba extract"[TW] OR "ginkgo extract"[TW] OR "ginkgold"[TW] OR "ginkgopower"[TW] OR "ginkobene"[TW] OR "ginkor"[TW] OR "kaveri"[TW] OR "kaveri forte"[TW] OR "li 1370"[TW] OR "superginkgo"[TW] OR "tanakene"[TW]
	#13	"Ginkgo biloba extract 50"[Supplementary Concept]
	#14	"EGB50 Ginkgo biloba extract"[TW] OR "GBE50"[TW]
	#15	"Ginkgo biloba extract VR456"[Supplementary Concept]

	#16	"VR456"[TW]
	#17	"Ginkgo biloba extract 501"[Supplementary Concept]
	#18	"EGb 501"[TW]
	#19	"Ginkgo biloba extract 1212"[Supplementary Concept]
	#20	"EGB1212"[TW]
	**#21**	**#8 OR #9 OR #10 OR #11 OR #12 OR #13 OR #14 OR #15 OR #16 OR #17 OR #18 OR #19 OR #20**
	**#22**	**#7 AND #21**
	#23	("Adaptive Clinical Trial" [PTYP:NoExp] OR "Clinical Trial" [PTYP:NoExp] OR "clinical trial, phase i"[PTYP] OR "clinical trial, phase ii"[PTYP] OR "clinical trial, phase iii"[PTYP] OR "clinical trial, phase iv"[PTYP] OR "controlled clinical trial"[PTYP] OR "Equivalence Trial"[PTYP] OR "multicenter study"[PTYP] OR "pragmatic clinical trial"[PTYP] OR "randomized controlled trial"[PTYP] OR "adaptive clinical trials as topic"[MeSH:noexp] OR "Clinical Studies as Topic"[mesh:noexp] OR "Clinical Trials as Topic"[mesh:noexp] OR "clinical trials, phase i as topic"[MeSH:noexp] OR "clinical trials, phase ii as topic"[MeSH:noexp] OR "clinical trials, phase iii as topic"[MeSH:noexp] OR "clinical trials, phase iv as topic"[MeSH:noexp] OR "controlled clinical trials as topic"[MeSH:noexp] OR "Double-Blind Method"[Mesh] OR "early termination of clinical trials"[MeSH:noexp] OR "Equivalence Trials as Topic"[MeSH:noexp] OR "Intention to Treat Analysis"[MeSH:noexp] OR "multicenter studies as topic"[MeSH:noexp] OR "Non-Randomized Controlled Trials as Topic"[MeSH:noexp] OR "Pragmatic Clinical Trials as Topic"[MeSH:noexp] OR "randomized controlled trials as topic"[MeSH:noexp] OR "2 arm"[TIAB] OR "two arm"[TIAB] OR "3 arm"[TIAB] OR "three arm"[TIAB] OR "4 arm"[TIAB] OR "four arm"[TIAB] OR "clinical studies"[TIAB:~3] OR "clinical study"[TIAB:~3] OR "clinical trial"[TIAB:~3] OR "clinical trials"[TIAB:~3] OR "controlled studies"[TIAB:~3] OR "controlled study"[TIAB:~3] OR "controlled trial"[TIAB:~3] OR "controlled trials"[TIAB:~3] OR ("cross-over"[TIAB] AND "over trial"[TIAB:~3]) OR ("cross-over"[TIAB] AND "over trials"[TIAB:~3]) OR "crossover trial"[TIAB:~3] OR "crossover trials"[TIAB:~3] OR "equivalent study"[TIAB:~3] OR "equivalent trial"[TIAB:~3] OR "equivalent trials"[TIAB:~3] OR intervention[TI] OR "pragmatic study"[TIAB:~3] OR "pragmatic trial"[TIAB:~3] OR "pragmatic trials"[TIAB:~3] OR "randomised cross"[TIAB] OR "randomised crossover"[TIAB] OR "randomized cross"[TIAB:~3] OR "randomized crossover"[TIAB:~3] OR "randomised studies"[TIAB:~3] OR "randomised study"[TIAB:~3] OR "randomised trial"[TIAB:~3] OR "randomised trials"[TIAB:~3] OR "randomized studies"[TIAB:~3] OR "randomized study"[TIAB:~3] OR "randomized trial"[TIAB:~3] OR "randomized trials"[TIAB:~3] OR "stepped wedge"[TIAB] OR ("window opportunity"[tiab:~1] AND trial[tiab]) OR "phase I"[TIAB] OR "phase II"[TIAB] OR "phase III"[TIAB] OR "phase IV"[TIAB] OR "phase 1"[TIAB] OR "phase 2"[TIAB] OR "phase 3"[TIAB] OR "phase 4"[TIAB] OR "single blind"[TIAB] OR "single blinded"[TIAB] OR "single mask"[TIAB] OR "single masked"[TIAB] OR "double blind"[TIAB] OR "double blinded"[TIAB] OR "double mask"[TIAB] OR "double masked"[TIAB] OR "triple blind"[TIAB] OR "triple blinded"[TIAB] OR "triple masked"[TIAB] OR "tripled blinded"[TIAB] OR "random assignment"[tiab] OR "randomly assigned"[TIAB]) NOT ((animals[mesh] OR plants[mesh]) NOT humans[mesh])
	**#24**	**#22 AND #23 AND (1980:2026[pdat])**

Database	No.	Search Query
**EMBASE**	#1	"intermittent claudication"/exp
	#2	"Intermittent Claudication":ti,ab,kw OR "intermittent arterial claudication":ti,ab,kw OR "intermittent claudicatio":ti,ab,kw OR "intermittent claudications":ti,ab,kw OR "intermittent vascular claudication":ti,ab,kw OR "leg ischaemia pain":ti,ab,kw OR "leg ischemia pain":ti,ab,kw OR "claudicant":ti,ab,kw OR "claudicants":ti,ab,kw
	#3	"peripheral arterial disease"/exp
	#4	"Peripheral Arterial Disease":ti,ab,kw OR "Peripheral Arterial Diseases":ti,ab,kw OR "Peripheral Artery Disease":ti,ab,kw OR "Peripheral Artery Diseases":ti,ab,kw OR "arterial obliterative disease":ti,ab,kw OR "arterial occlusive diseases":ti,ab,kw OR "artery chronic occlusive disease":ti,ab,kw OR "artery obliterative disease":ti,ab,kw OR "artery occlusive disease":ti,ab,kw OR "chronic arterial occlusion disease":ti,ab,kw OR "chronic artery obstruction":ti,ab,kw OR "chronic artery occlusion":ti,ab,kw OR "obliterative arterial disease":ti,ab,kw OR "obliterative artery disease":ti,ab,kw OR "obstructive arterial disease":ti,ab,kw OR "obstructive artery disease":ti,ab,kw OR "occlusive arterial disease":ti,ab,kw OR "occlusive artery disease":ti,ab,kw OR "PAD":ti,ab,kw OR "PAOD":ti,ab,kw OR "peripheral arterial disorder":ti,ab,kw OR "peripheral arterial obstructive disease":ti,ab,kw OR "peripheral artery obstructive disease":ti,ab,kw OR "peripheral atherosclerosis":ti,ab,kw OR "peripheral obliterative arterial disease":ti,ab,kw OR "peripheral obliterative vascular disease":ti,ab,kw OR "peripheral obstructive artery disease":ti,ab,kw OR "peripheral occlusive arterial disease":ti,ab,kw OR "peripheral occlusive artery disease":ti,ab,kw OR "peripheral occlusive disease":ti,ab,kw OR "peripheral vascular occlusive disease":ti,ab,kw
	#5	"peripheral arterial occlusive disease":ti,ab,kw OR "peripheral arterial occlusive diseases":ti,ab,kw OR "Fontaine stage II":ti,ab,kw OR "Rutherford stage":ti,ab,kw
	**#6**	**#1 OR #2 OR #3 OR #4 OR #5**
	#7	"Ginkgo biloba"/exp
	#8	"Ginkgo biloba":ti,ab,kw OR "Ginkgo bilobas":ti,ab,kw OR "Gingko":ti,ab,kw OR "Gingkos":ti,ab,kw OR "Gingko biloba":ti,ab,kw OR "Gingko bilobas":ti,ab,kw OR "Ginkgo":ti,ab,kw OR "Ginkgos":ti,ab,kw OR "Ginko":ti,ab,kw OR "Ginkos":ti,ab,kw
	#9	"Ginkgo biloba extract"/exp
	#10	"Ginkgo leaf extract":ti,ab,kw OR "Tebofortran":ti,ab,kw OR "Tebokan":ti,ab,kw OR "Tebonin":ti,ab,kw OR "EGb-761":ti,ab,kw OR "EGb761":ti,ab,kw OR "GBE 761 ONC":ti,ab,kw OR "Rokan":ti,ab,kw OR "Tanakan":ti,ab,kw OR "GBE-761":ti,ab,kw OR "Ginkgo biloba extract 761":ti,ab,kw OR "gingko biloba extract":ti,ab,kw OR "ginkgo extract":ti,ab,kw OR "ginkgold":ti,ab,kw OR "ginkgopower":ti,ab,kw OR "ginkobene":ti,ab,kw OR "ginkor":ti,ab,kw OR "kaveri":ti,ab,kw OR "kaveri forte":ti,ab,kw OR "li 1370":ti,ab,kw OR "superginkgo":ti,ab,kw OR "tanakene":ti,ab,kw
	#11	"Ginkgo biloba extract 50":ti,ab,kw OR "EGB50 Ginkgo biloba extract":ti,ab,kw OR "GBE50":ti,ab,kw
	#12	"Ginkgo biloba extract VR456":ti,ab,kw
	#13	"VR456":ti,ab,kw
	#14	"Ginkgo biloba extract 501":ti,ab,kw

	#15	"EGb 501":ti,ab,kw
	#16	"Ginkgo biloba extract 1212":ti,ab,kw
	#17	"EGB1212":ti,ab,kw
	**#18**	**#7 OR #8 OR #9 OR #10 OR #11 OR #12 OR #13 OR #14 OR #15 OR #16 OR #17**
	**#19**	**#6 AND #18**
	#20	#19 AND [randomized controlled trial]/lim
	#21	#19 AND ('randomized controlled trial (topic)'/exp OR 'randomized controlled trial':it OR 'randomization'/exp OR 'double blind procedure'/exp OR 'single blind procedure'/exp OR 'clinical trial':it OR 'clinical trial, phase i':it OR 'clinical trial, phase ii':it OR 'clinical trial, phase iii':it OR 'clinical trial, phase iv':it OR 'controlled clinical trial':it OR 'multicenter study':it OR 'clinical trial (topic)'/exp OR ('clinical':ti,ab AND 'trial*':ti,ab) OR (('singl*':ti,ab OR 'doubl*':ti,ab OR 'treb*':ti,ab OR 'tripl*':ti,ab) AND ('blind*':ti,ab OR 'mask*':ti,ab)) OR 'placebo'/exp OR 'placebo*':ti,ab OR 'randomly allocated':ti,ab OR ('allocated':ti,ab AND 'random*':ti,ab)) NOT ('case report':ti,ab OR 'letter':it OR 'historical article':it)
	#22	#20 OR #21
	#23	#22 NOT (('animal'/exp OR 'plant'/exp) NOT 'human'/exp) OR (#22 NOT 'comparative study'/exp)
	**#24**	**#23 AND [1980-2026]/py**

Database	No.	Search Query
**Cochrane Library**	#1	[mh "Intermittent Claudication"]
	#2	"Intermittent Claudication":ti,ab,kw OR "intermittent arterial claudication":ti,ab,kw OR "intermittent claudicatio":ti,ab,kw OR "intermittent claudications":ti,ab,kw OR "intermittent vascular claudication":ti,ab,kw OR "leg ischaemia pain":ti,ab,kw OR "leg ischemia pain":ti,ab,kw OR "claudicant":ti,ab,kw OR "claudicants":ti,ab,kw
	#3	[mh "Peripheral Arterial Disease"]
	#4	"Peripheral Arterial Disease":ti,ab,kw OR "Peripheral Arterial Diseases":ti,ab,kw OR "Peripheral Artery Disease":ti,ab,kw OR "Peripheral Artery Diseases":ti,ab,kw OR "arterial obliterative disease":ti,ab,kw OR "arterial occlusive diseases":ti,ab,kw OR "artery chronic occlusive disease":ti,ab,kw OR "artery obliterative disease":ti,ab,kw OR "artery occlusive disease":ti,ab,kw OR "chronic arterial occlusion disease":ti,ab,kw OR "chronic artery obstruction":ti,ab,kw OR "chronic artery occlusion":ti,ab,kw OR "obliterative arterial disease":ti,ab,kw OR "obliterative artery disease":ti,ab,kw OR "obstructive arterial disease":ti,ab,kw OR "obstructive artery disease":ti,ab,kw OR "occlusive arterial disease":ti,ab,kw OR "occlusive artery disease":ti,ab,kw OR "PAD":ti,ab,kw OR "PAOD":ti,ab,kw OR "peripheral arterial disorder":ti,ab,kw OR "peripheral arterial obstructive disease":ti,ab,kw OR "peripheral artery obstructive disease":ti,ab,kw OR "peripheral atherosclerosis":ti,ab,kw OR "peripheral obliterative arterial disease":ti,ab,kw OR "peripheral obliterative vascular disease":ti,ab,kw OR "peripheral obstructive artery disease":ti,ab,kw OR "peripheral occlusive arterial disease":ti,ab,kw OR "peripheral occlusive artery disease":ti,ab,kw OR "peripheral occlusive disease":ti,ab,kw OR "peripheral vascular occlusive disease":ti,ab,kw

	#5	"peripheral arterial occlusive disease":ti,ab,kw OR "peripheral arterial occlusive diseases":ti,ab,kw OR "Fontaine stage II":ti,ab,kw OR "Rutherford stage":ti,ab,kw
	**#6**	**#1 OR #2 OR #3 OR #4 OR #5**
	#7	[mh "Ginkgo biloba"]
	#8	"Ginkgo biloba":ti,ab,kw OR "Ginkgo bilobas":ti,ab,kw OR "Gingko":ti,ab,kw OR "Gingkos":ti,ab,kw OR "Gingko biloba":ti,ab,kw OR "Gingko bilobas":ti,ab,kw OR "Ginkgo":ti,ab,kw OR "Ginkgos":ti,ab,kw OR "Ginko":ti,ab,kw OR "Ginkos":ti,ab,kw
	#9	[mh "Ginkgo Extract"]
	#10	"Ginkgo leaf extract":ti,ab,kw OR "Tebofortran":ti,ab,kw OR "Tebokan":ti,ab,kw OR "Tebonin":ti,ab,kw OR "EGb-761":ti,ab,kw OR "EGb761":ti,ab,kw OR "GBE 761 ONC":ti,ab,kw OR "Rokan":ti,ab,kw OR "Tanakan":ti,ab,kw OR "GBE-761":ti,ab,kw OR "Ginkgo biloba extract 761":ti,ab,kw OR "gingko biloba extract":ti,ab,kw OR "ginkgo extract":ti,ab,kw OR "ginkgold":ti,ab,kw OR "ginkgopower":ti,ab,kw OR "ginkobene":ti,ab,kw OR "ginkor":ti,ab,kw OR "kaveri":ti,ab,kw OR "kaveri forte":ti,ab,kw OR "li 1370":ti,ab,kw OR "superginkgo":ti,ab,kw OR "tanakene":ti,ab,kw
	#11	"Ginkgo biloba extract 50":ti,ab,kw
	#12	"EGB50 Ginkgo biloba extract":ti,ab,kw OR "GBE50":ti,ab,kw
	#13	"Ginkgo biloba extract VR456":ti,ab,kw
	#14	"VR456":ti,ab,kw
	#15	"Ginkgo biloba extract 501":ti,ab,kw
	#16	"EGb 501":ti,ab,kw
	#17	"Ginkgo biloba extract 1212":ti,ab,kw
	#18	"EGB1212":ti,ab,kw
	**#19**	**#7 OR #8 OR #9 OR #10 OR #11 OR #12 OR #13 OR #14 OR #15 OR #16 OR #17 OR #18**
	**#20**	**#6 AND #19**
	#21	#20 NOT (([mh "animals"] OR [mh "plants"]) NOT [mh "humans"]) OR (#20 NOT [mh "comparative study"])
	**#22**	**#21**<br>**with Cochrane Library publication date from Jan 1980 to Dec 2026**

Database	No.	Search Query
**CINAHL**	S1	MH "Intermittent Claudication"
	S2	TX ("Intermittent Claudication" OR "intermittent arterial claudication" OR "intermittent claudicatio" OR "intermittent claudications" OR "intermittent vascular claudication" OR "leg ischaemia pain" OR "leg ischemia pain" OR "claudicant" OR "claudicants")
	S3	MH "Peripheral Vascular Diseases"

	S4	TX ("Peripheral Arterial Disease" OR "Peripheral Arterial Diseases" OR "Peripheral Artery Disease" OR "Peripheral Artery Diseases" OR "arterial obliterative disease" OR "arterial occlusive diseases" OR "artery chronic occlusive disease" OR "artery obliterative disease" OR "artery occlusive disease" OR "chronic arterial occlusion disease" OR "chronic artery obstruction" OR "chronic artery occlusion" OR "obliterative arterial disease" OR "obliterative artery disease" OR "obstructive arterial disease" OR "obstructive artery disease" OR "occlusive arterial disease" OR "occlusive artery disease" OR "PAD" OR "PAOD" OR "peripheral arterial disorder" OR "peripheral arterial obstructive disease" OR "peripheral artery obstructive disease" OR "peripheral atherosclerosis" OR "peripheral obliterative arterial disease" OR "peripheral obliterative vascular disease" OR "peripheral obstructive artery disease" OR "peripheral occlusive arterial disease" OR "peripheral occlusive artery disease" OR "peripheral occlusive disease" OR "peripheral vascular occlusive disease")
	S5	TX ("peripheral arterial occlusive disease" OR "peripheral arterial occlusive diseases" OR "Fontaine stage II" OR "Rutherford stage")
	**S6**	**S1 OR S2 OR S3 OR S4 OR S5**
	S7	MH "Ginkgo Biloba"
	S8	TX ("Ginkgo biloba" OR "Ginkgo bilobas" OR "Gingko" OR "Gingkos" OR "Gingko biloba" OR "Gingko bilobas" OR "Ginkgo" OR "Ginkgos" OR "Ginko" OR "Ginkos")
	S9	TX ("Ginkgo Extract")
	S10	TX ("Ginkgo leaf extract" OR "Tebofortran" OR "Tebokan" OR "Tebonin" OR "EGb-761" OR "EGb761" OR "GBE 761 ONC" OR "Rokan" OR "Tanakan" OR "GBE-761" OR "Ginkgo biloba extract 761" OR "gingko biloba extract" OR "ginkgo extract" OR "ginkgold" OR "ginkgopower" OR "ginkobene" OR "ginkor" OR "kaveri" OR "kaveri forte" OR "li 1370" OR "superginkgo" OR "tanakene")
	S11	TX ("Ginkgo biloba extract 50")
	S12	TX ("EGB50 Ginkgo biloba extract" OR "GBE50")
	S13	TX ("Ginkgo biloba extract VR456")
	S14	TX ("VR456")
	S15	TX ("Ginkgo biloba extract 501")
	S16	TX ("EGb 501")
	S17	TX ("Ginkgo biloba extract 1212")
	S18	TX ("EGB1212")
	**S19**	**S7 OR S8 OR S9 OR S10 OR S11 OR S12 OR S13 OR S14 OR S15 OR S16 OR S17 OR S18**
	**S20**	**S6 AND S19**
	S21	S20 AND MH "Randomized Controlled Trials+"
	S22	S21 NOT ((MH "Animals+" OR MH "Plants+") NOT MH "Human") OR (S20 NOT MH "Comparative Studies+")
	S23	S21 OR S22

	S24	S23<br>01/01/1980 - 12/31/2026

Supplementary Table S4. Adverse events

Study	Adverse events
Bauer 1984 [1]	Placebo: weakness (n=1) and stomach disorder (n=1). Ginkgo: nausea and blood in urine were reported; nausea occurred during the placebo run-in period, and blood in urine was reported at Week 24 in a patient with bladder cancer. No clinically relevant effects on blood pressure or heart rate were observed.
Blume 1996 [2]	Two non-serious unintended events occurred in the Ginkgo group (stomach pain, n=1; nausea, n=1), compared with none in the placebo group. Both resolved during treatment and were considered unlikely to be causally related to treatment.
Blume 1998 [3]	No adverse drug reactions were observed during treatment.
Bulling 1991 [4]	No adverse events or unintended effects were observed.
Diehm 1990 [5]	No side effects were observed.
Natali 1985 [6]	Adverse reaction(s) were reported. The Cochrane review includes this study among four trials reporting nausea, gastrointestinal complaints, hematuria, or headache; however, the review does not specify the individual adverse-event type attributable to Natali 1985.
Drabaek 1996 [7]	Two participants discontinued the study: one because of severe dyspnea during active treatment and one because of a transient cerebral ischemic attack, which was attributed to withdrawal of acetylsalicylic acid. One participant reported increased hair growth during active treatment but completed the study. No significant changes in laboratory safety parameters were observed.
Peters 1998 [8]	No adverse events occurred in the Ginkgo group. One placebo-treated participant reported heartburn and gastric pain. No discontinuations were attributed to side effects.
Schoop 1997 [9]	No side effects were observed.
Thomson 1990 [10]	No major clinical, hematological, or biochemical side effects were observed, and clinical and biological tolerability did not differ between groups. One placebo-treated participant withdrew because of dizziness.
Kim 2026 [11]	The overall incidence of adverse events was similar between the GNX80 and placebo groups (35.1% vs 26.2%, respectively; P = 0.296). No significant between-group differences were observed in the incidence of adverse events across individual system organ classes.

Supplementary Table S5. Sensitivity analysis

Analysis	Outcome	No. of studies	Participants (T/C)	Mean difference, kcal (95% CI)
Exclusion of discrepant study [12]	ACD	11	288/279	4.42 (2.31 to 6.53)
Exclusion of high-risk studies [10]	ACD	12	296/288	3.50 (0.02 to 6.98)
Exclusion of one large trial [11]	ACD (24w)	6	166/155	4.72 (2.27 to 7.17)

ACD, Absolute Claudication Distance; CI, Confidence Interval; T, Ginkgo biloba Group; C, Placebo Group.

Supplementary Table S6. GRADE assessment

Certainty assessment							№ of patients		Effect	Certainty	Importance
№ of studies	Study design	Risk of bias	Inconsistency	Indirectness	Imprecision	Other considerations	Ginkgo biloba extract	Placebo	Absolute (95% CI)		
**Absolute claudication distance (ACD) (follow-up: range 6 weeks to 24 weeks; assessed with: Treadmill walking test)**											
12	randomised trials	not serious	Serious*	not serious	not serious	none	296	288	MD 3.50 Kcal higher (0.02 higher to 6.98 higher)	⊕⊕⊕◯ Moderate	CRITICAL
**Initial claudication distance (ICD) (follow-up: range 6 weeks to 24 weeks; assessed with: Treadmill walking test)**											
14	randomised trials	not serious	Serious*	not serious	serious†	none	423	407	MD 1.89 Kcal higher (0.80 lower to 4.58 higher)	⊕⊕◯◯ Low	IMPORTANT
**ABI and ankle pressure, end of study (follow-up: range 12 weeks to 24 weeks; assessed with: Doppler-measured ankle systolic pressure and ankle-brachial index (ABI))**											
7	randomised trials	not serious	not serious	not serious	serious†	none	194	187	SMD 0.17 lower (0.47 lower to 0.12 higher)	⊕⊕⊕◯ Moderate	IMPORTANT
**Adverse events (follow-up: range 12 weeks to 24 weeks; assessed with: Adverse events, side effects, and clinical or biological tolerability reported by individual trials)**											
11	randomised trials	not serious	not serious	not serious	serious†	none			Narrative review	⊕⊕⊕◯ Moderate	IMPORTANT

CI, Confidence Interval; GRADE, Grading of Recommendations Assessment Development and Evaluation; MD, Mean Difference; SMD, Standardized Mean Difference.

*Downgraded one level for inconsistency due to statistical heterogeneity and variability in size of effect

†Downgraded one level for imprecision due to confidence intervals spanning the null effect and appreciably different effect estimates.

Study	Ginkgo biloba N	Ginkgo biloba Mean	Ginkgo biloba SD	Placebo N	Placebo Mean	Placebo SD	Weight	Mean Difference IV, Random, 95% CI
Bauer 1984	44	14.0	13.2	34	12.3	10.9	6.8%	1.70 [-3.65, 7.05]
Natali 1985	9	12.1	4.3	9	10.0	7.6	6.6%	2.10 [-3.60, 7.80]
Thomson 1990	20	13.2	10.0	17	14.9	10.4	6.0%	-1.70 [-8.31, 4.91]
Diehm 1990	22	18.4	5.6	18	16.7	6.6	7.9%	1.70 [-2.14, 5.54]
Bulling 1991	17	27.0	11.0	16	18.8	13.1	5.0%	8.20 [-0.08, 16.48]
Blume 1996	28	22.3	7.0	26	17.7	4.3	8.4%	4.60 [1.53, 7.67]
Drabaek 1996	9	14.1	5.1	9	14.3	5.2	7.3%	-0.20 [-4.96, 4.56]
Peters 1998	47	19.5	4.5	52	16.4	11.2	8.2%	3.10 [-0.20, 6.40]
Blume 1998	21	17.4	8.2	19	11.4	5.8	7.5%	6.00 [1.63, 10.37]
Wang 2007	8	5.8	2.6	9	8.0	4.3	8.2%	-2.20 [-5.54, 1.14]
Gardner 2008	30	8.9	4.8	28	8.5	6.1	8.5%	0.40 [-2.44, 3.24]
Salz 1980	13	20.3	7.4	13	11.0	7.5	6.6%	9.30 [3.57, 15.03]
Schoop 1997	105	17.8	4.6	100	22.9	4.8	9.2%	-5.10 [-6.39, -3.81]
Kim 2026	50	35.3	28.4	57	32.2	25.9	3.9%	3.07 [-7.28, 13.41]
**Random effects model**	**423**			**407**			**100.0%**	**1.89 [-0.80, 4.58]**

Heterogeneity: I^2 = 85.7%, τ^2 = 20.1461, p < 0.0001

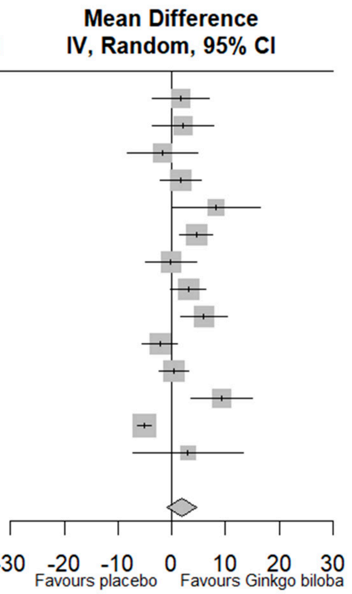

Supplementary Figure S1. Forest plot of initial claudication distance (expressed as kilocalories) at the end of the study: Ginkgo biloba versus placebo

CI, confidence interval; IV, inverse variance; SD, standard deviation; I², percentage of variability due to between-study heterogeneity; τ², between-study variance.

Study	Ginkgo biloba N	Ginkgo biloba Mean	Ginkgo biloba SD	Placebo N	Placebo Mean	Placebo SD	Weight	Mean Difference IV, Random, 95% CI
Bauer 1984	44	14.0	13.2	34	12.3	10.9	11.2%	1.70 [-3.65, 7.05]
Natali 1985	9	12.1	4.3	9	10.0	7.6	10.9%	2.10 [-3.60, 7.80]
Thomson 1990	20	13.2	10.0	17	14.9	10.4	10.2%	-1.70 [-8.31, 4.91]
Bulling 1991	17	27.0	11.0	16	18.8	13.1	8.8%	8.20 [-0.08, 16.48]
Blume 1996	28	22.3	7.0	26	17.7	4.3	13.0%	4.60 [1.53, 7.67]
Peters 1998	47	19.5	4.5	52	16.4	11.2	12.8%	3.10 [-0.20, 6.40]
Blume 1998	21	17.4	8.2	19	11.4	5.8	12.0%	6.00 [1.63, 10.37]
Schoop 1997	105	17.8	4.6	100	22.9	4.8	13.8%	-5.10 [-6.39, -3.81]
Kim 2026	50	35.3	28.4	57	32.2	25.9	7.3%	3.07 [-7.28, 13.41]
**Random effects model**	**341**			**330**			**100.0%**	**2.20 [-1.81, 6.21]**

Heterogeneity: $I^2 = 89.0\%$, $\tau^2 = 29.8332$, $p < 0.0001$

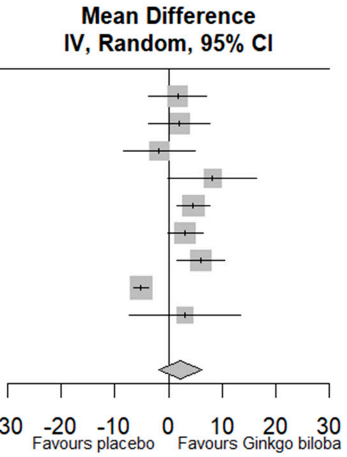

Supplementary Figure S2. Forest plot of initial claudication distance (expressed as kilocalories) at 24 weeks: Ginkgo biloba versus placebo

CI, confidence interval; IV, inverse variance; SD, standard deviation; I², percentage of variability due to between-study heterogeneity; τ², between-study variance.

Study	Ginkgo biloba N	Ginkgo biloba Mean	Ginkgo biloba SD	Placebo N	Placebo Mean	Placebo SD	Weight	Mean Difference IV, Random, 95% CI
Bauer 1984	44	12.3	14.7	35	10.5	6.9	7.1%	1.80 [-3.11, 6.71]
Diehm 1990	22	18.4	5.6	18	16.7	6.6	10.0%	1.70 [-2.14, 5.54]
Bulling 1991	17	16.3	6.5	16	18.8	13.9	3.5%	-2.50 [-9.98, 4.98]
Blume 1996	29	22.1	7.7	28	17.7	5.1	11.8%	4.40 [1.02, 7.78]
Drabaek 1996	9	14.1	5.1	9	14.3	5.2	7.4%	-0.20 [-4.96, 4.56]
Peters 1998	47	17.9	4.0	52	15.8	5.7	19.7%	2.10 [0.17, 4.03]
Blume 1998	21	15.8	5.7	19	10.7	5.4	11.5%	5.10 [1.66, 8.54]
Wang 2007	8	5.8	2.6	9	8.0	4.3	12.0%	-2.20 [-5.54, 1.14]
Gardner 2008	30	8.9	4.8	28	8.5	6.1	14.3%	0.40 [-2.44, 3.24]
Kim 2026	50	28.3	22.6	57	28.6	24.1	2.6%	-0.25 [-9.10, 8.60]
**Random effects model**	**277**			**271**			**100.0%**	**1.50 [-0.00, 3.01]**

Heterogeneity: $I^2 = 37.6\%$, $\tau^2 = 2.0226$, $p = 0.1083$

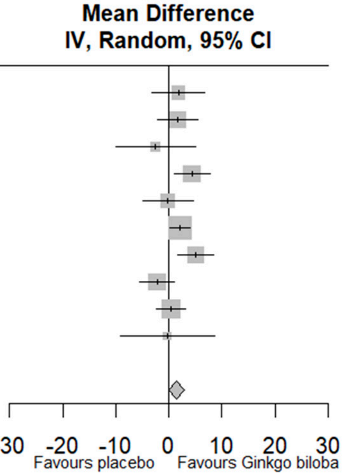

Supplementary Figure S3. Forest plot of initial claudication distance (expressed as kilocalories) at 12–16 weeks: Ginkgo biloba versus placebo

CI, confidence interval; IV, inverse variance; SD, standard deviation; I², percentage of variability due to between-study heterogeneity; τ², between-study variance.

Study	Ginkgo biloba N	Ginkgo biloba Mean	Ginkgo biloba SD	Placebo N	Placebo Mean	Placebo SD	Weight	Std. Mean Difference IV, Random, 95% CI
Bauer 1984	44	87.40	23.90	35	78.60	20.70	17.3%	0.39 [-0.06, 0.83]
Bulling 1991 (Posterior tibial)	17	79.70	21.80	16	97.30	30.10	10.9%	-0.66 [-1.36, 0.05]
Bulling 1991 (Dorsalis pedis)	17	77.20	18.25	16	93.30	28.00	10.9%	-0.67 [-1.37, 0.03]
Drabaek 1996	9	0.53	0.20	9	0.57	0.24	7.5%	-0.17 [-1.10, 0.75]
Gardner 2008	29	0.79	0.28	28	0.76	0.19	15.2%	0.12 [-0.40, 0.64]
Thomson 1990	20	90.50	21.10	17	103.50	18.30	11.7%	-0.64 [-1.30, 0.02]
Wang 2007	8	0.62	0.16	9	0.67	0.19	7.1%	-0.27 [-1.23, 0.69]
Kim 2026	50	0.73	0.19	57	0.74	0.21	19.5%	-0.05 [-0.43, 0.33]
**Random effects model**	**194**			**187**			**100.0%**	**-0.17 [-0.47, 0.12]**

Heterogeneity: $I^2 = 45.7\%$, $\tau^2 = 0.0770$, $p = 0.0746$

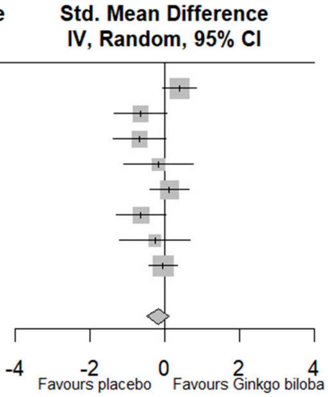

Supplementary Figure S4. Forest plot of ABI & ankle pressure at the end of the study: Ginkgo biloba versus placebo

CI, confidence interval; IV, inverse variance; SD, standard deviation; I², percentage of variability due to between-study heterogeneity; τ², between-study variance.

Peters 1998 reported group-specific Doppler index values at baseline and study end but no measures of variability, precluding inclusion in the Cochrane quantitative analysis of ABI/ankle pressure.

Blume 1996 reported the Doppler index as an identical combined median value at baseline and study end, without group-specific values or measures of variability, precluding inclusion in the Cochrane quantitative analysis of ABI/ankle pressure.

Blume 1998 reported group-specific baseline ankle pressure values but no study-end values or measures of variability, precluding inclusion in the Cochrane quantitative analysis of ABI/ankle pressure.

(a)

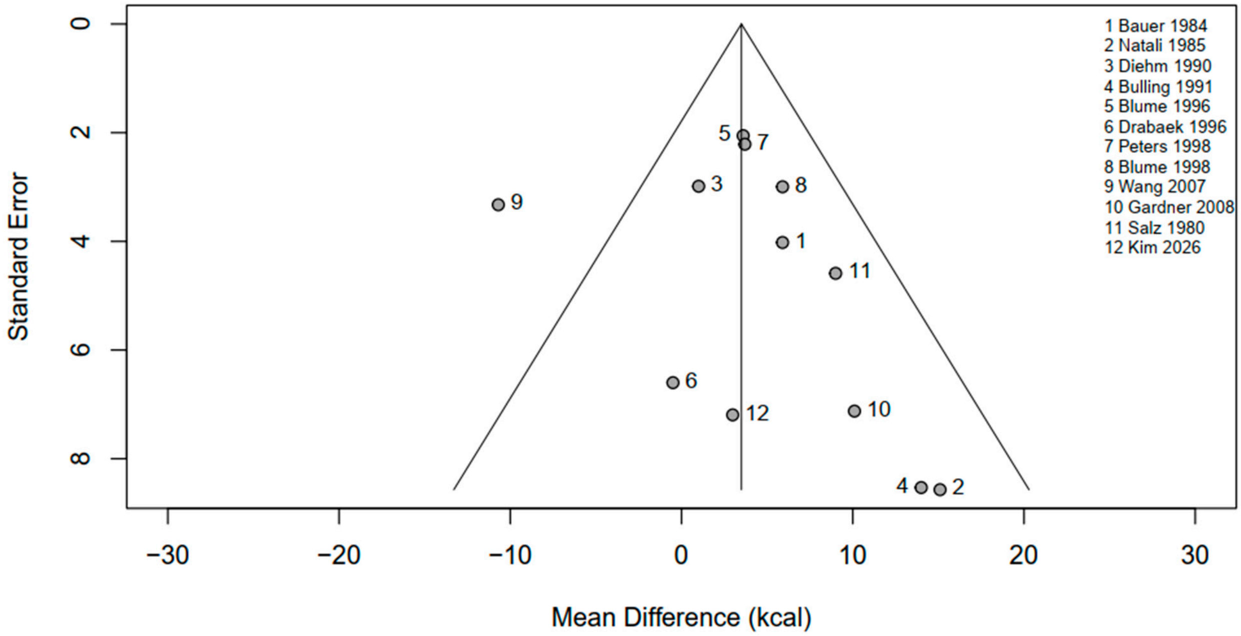

(b)

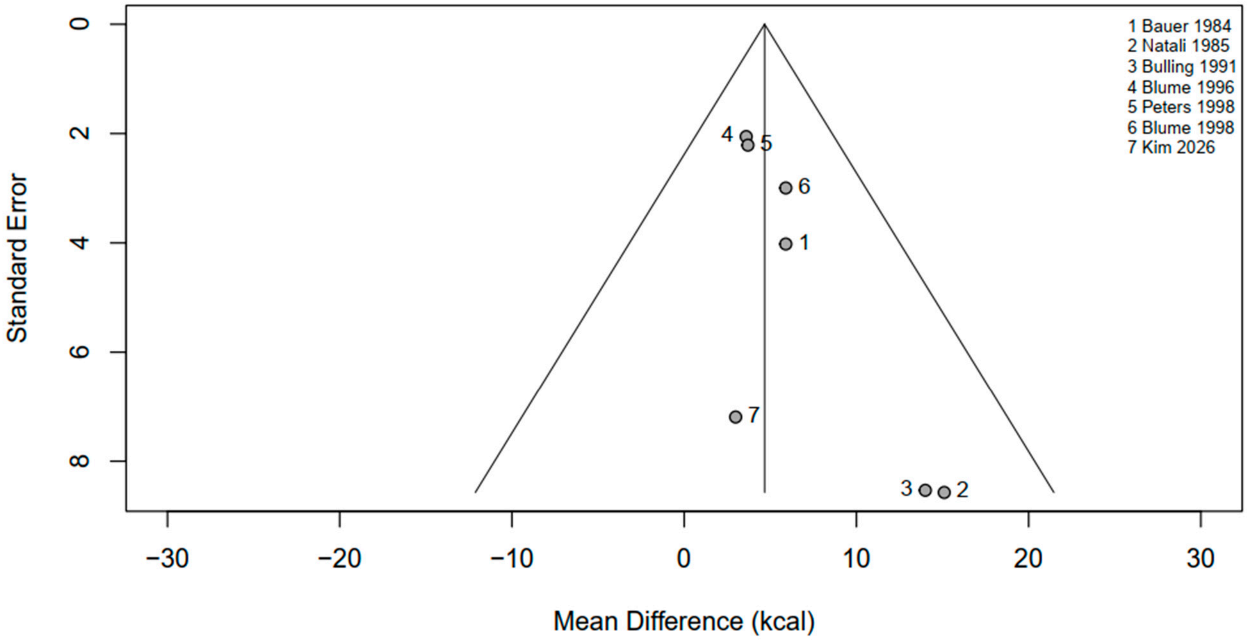

Supplementary Figure S5. Funnel plots for ACD

(a) At the end of study; (b) At 24 weeks.

REFERENCES

1. Bauer, U. 6-Month double-blind randomised clinical trial of Ginkgo biloba extract versus placebo in two parallel groups in patients suffering from peripheral arterial insufficiency. *Arzneimittelforschung* **1984**, *34*, 716–720.
2. Blume, J.; Kieser, M.; Hölscher, U. Placebo-controlled double-blind study of the effectiveness of Ginkgo biloba special extract EGb 761 in trained patients with intermittent claudication. *Vasa* **1996**, *25*, 265–274.
3. Blume, J.; Kieser, M.; Hölscher, U. Ginkgo-special-extract EGb 761 in peripheral arterial occlusive diseases stage IIb according to Fontaine. *Fortschr Med* **1998**, *116*, 36–37.
4. Bulling, B.; Vonbary, S. Treatment of chronic peripheral occlusive disease with physical-training and Ginkgo-biloba excract 761. *Medizinische Welt* **1991**, *42*, 702–708.
5. Diehm C, H.F., Mörl H. Placebo-controlled, multicentre, randomised, double-blind pilot study examining the effectiveness of Ginkgo biloba extract EGb 761® in patients with Fontaine stage IIb peripheral arterial disease. *Internal report, Dr. Willmar Schwabe GmbH & Co.* **1990**.
6. Natali, J. Protocol for a study comparing Ginkgo biloba extract and placebo in patients with arteriopathy of the lower extremities. *Internal Study Report, Ipsen, Paris* **1985**.
7. Drabaek, H.; Petersen, J.R.; Winberg, N.; Hansen, K.F.; Mehlsen, J. The effect of Ginkgo biloba extract in patients with intermittent claudication. *Ugeskr Laeger* **1996**, *158*, 3928–3931.
8. Peters, H.; Kieser, M.; Hölscher, U. Demonstration of the efficacy of ginkgo biloba special extract EGb 761 on intermittent claudication--a placebo-controlled, double-blind multicenter trial. *Vasa* **1998**, *27*, 106–110.
9. Schoop, W.; Breddin, K.; Diehm, C.; Gruß, J.; Held, K. Klinische Prüfung mit Ginkgo biloba-Spezialextrakt Egb 761 bei Patienten mit peripherer arterieller Verschlußkrankheit im Stadium II b nach Fontaine im Vergleich zu Placebo. *Posterpublikation, Jahreskongress der Schweizerischen Gesellschaft für Angiologie* **1996**, *1*, 1996.
10. Thomson, G.; Vohra, R.; Carr, M.; Walker, M. A clinical trial of Gingkco biloba extract in patients with intermittent claudication. *International angiology: a journal of the International Union of Angiology* **1990**, *9*, 75–78.
11. Kim, O.; Lee, S.H.; Kim, J.Y.; Kim, W.; Lee, H.C.; Roh, J.W.; Joh, J.H.; Park, S.-H.; Shin, S.; Park, S.-H.; et al. Efficacy and Safety of GNX80 in Patients with Peripheral Arterial

Disease and Intermittent Claudication. **2026**.

12. Wang, J.; Zhou, S.; Bronks, R.; Graham, J.; Myers, S. Supervised exercise training combined with ginkgo biloba treatment for patients with peripheral arterial disease. *Clin Rehabil* **2007**, *21*, 579–586, doi:10.1177/0269215507075205.
